# The Complete Chloroplast Genome of *Curcuma bakerii*, an Endemic Medicinal Plant of Bangladesh: Insights into Genome Structure, Comparative Genomics, and Phylogenetic Relationships

**DOI:** 10.3390/genes16121460

**Published:** 2025-12-07

**Authors:** Mohammad Rashedul Islam, Dhafer A. Alzahrani, Enas J. Albokhari, Mohammad S. Alawfi, Arwa I. Alsubhi

**Affiliations:** 1 Department of Biological Sciences, Faculty of Sciences, King Abdulaziz University, Jeddah 21589, Saudi Arabia; dalzahrani@kau.edu.sa; 2 Department of Biology, Faculty of Sciences, Umm Al-Qura University, Makkah 24381, Saudi Arabia; ejbokhary@uqu.edu.sa; 3 Department of Biology, College of Sciences, King Khalid University, Abha 61421, Saudi Arabia; malawfi@kku.edu.sa; 4 Department of Biology, College of Science, Taibah University, Madinah 42353, Saudi Arabia; asubhia@taibahu.edu.sa

**Keywords:** *Curcuma bakerii*, endemic, Bangladesh, chloroplast (cp) genome, polymorphisms, phylogeny

## Abstract

**Background**: *Curcuma bakerii* is a species of the family Zingiberaceae, endemic to Bangladesh. This genus of rhizomatous plants is widely distributed in tropical regions worldwide and is valued for its medicinal, aromatic, and culinary properties. **Methods**: The complete chloroplast (cp) genome of *C. bakerii* was reconstructed using high-throughput sequencing data. Subsequently, the genome was functionally annotated, assembled, and analyzed to clarify its evolutionary dynamics and structural organization. **Results**: The study’s findings indicate that the genome size is 162,189 base pairs (bp) and that it has a normal quadripartite structure with a large single-copy (LSC) region also comprises a small single-copy (SSC) region and two inverted repeats (IRa and IRb). The GC content of the genome was 36.18%, consisting of 135 genes: 88 protein-coding, 39 tRNA, and 8 rRNA. The codon usage analysis revealed 22 high-frequency and five optimal codons indicative of codon bias. Analysis of repetitive sequences revealed 213 Simple Sequence Repeats (SSRs), most of which were A/T. Additionally, seven mutation hotspots were reported, with 68.08% of single-nucleotide polymorphisms (SNPs) detected in the coding region and 31.91% in the noncoding region. Nonsynonymous substitutions accounted for 63.78%, while synonymous substitutions accounted for 36.11%. **Conclusions**: Based on this study, cp genome sequencing is a useful tool for understanding the intrageneric relationships among *Curcuma* species. The research presents a complete cp genome of *C. bakerii* from Bangladesh and provides a useful genomic resource for the molecular evolution, phylogeny, and genetic diversity study of the genus *Curcuma*.

## 1. Introduction

The ginger family, or Zingiberaceae, is the largest and most diverse family in the order Zingiberales [1,2]. There are over 50 genera and about 1300 species, predominantly spread out in tropical and subtropical areas [3,4,5]. The perennial herb *Curcuma*, which belongs to this family, is native to tropical Asia, Australia, and the South Pacific [6,7]. Plants with rhizomes are common sources of food, medicine, and economic value [8,9]. In Asia, we can find them as spices, natural dyes, and traditional medicines. Research using phenotype screening has identified various curcuma species with antioxidant, antidiabetic, antitumor, neuroprotective, hepatoprotective, and antimicrobial properties [10]. The Family Zingiberaceae contains several useful species. The Zingiberaceae family contains several species valued for their economic and medicinal uses. Among them, about 50 *Curcuma* species are native to Southeast Asia [11], and nine species have been recorded in the flora of Bangladesh [12,13]. Those plants are rhizomatous herbs that typically grow in shaded forests [14].

Different methods have been utilised for the accurate identification of *Curcuma* species. Morphological traits have been used in developing taxonomical keys, especially the inflorescence structures [15,16]. The inflorescence is especially useful in distinguishing the *Curcuma* species [17]. However, the inflorescences of these species often fail to reach full development due to short, irregular blooming periods. However, the *Curcuma* species are dormant in winter, with fleshy rhizomes and tuberous roots that normally flower in the rainy season and exhibit a mechanism similar to that of drought-resistant plants. Knowledge of evolutionary history can help understand species-environment interactions and apply it to enhance plant productivity [18]. The limited availability of inflorescences complicates the differentiation between several *Curcuma* species [16]. Molecular approaches are needed because morphological characters used for delimiting *Curcuma* lineages did not help clarify some closely related species [19]. Since nuclear reference genomes remain limited and hybrid or mixed lines are common in *Curcuma*, plastome-based approaches are among the most effective methods for resolving taxonomic relationships in plants [20].

Due to recent advances in high-throughput sequencing, whole cp genome records have become plentiful [21]. These resources will be valuable for investigating genetic markers and gene expression variability [22], as well as for inferring phylogenetic relationships and adaptive evolution both within and among angiosperms [23]. The evolution of taxa under selection pressures provides important insights. Multiple genes that have been positively selected for have been discovered in previous studies [24,25]. Traditional DNA barcoding markers are effective for identifying different species, but they often lack sufficient resolution to differentiate closely related taxa. Cp genome sequencing has emerged as a powerful tool that has successfully discriminated between closely related species of *Boesenbergia*, *Curcuma*, *Kaempferia* and *Pyrgophyllum* [26].

In plants, chloroplasts play an essential role and are unique because they carry their own genetic material [27,28]. Plants absorb sunlight and convert it into glucose via photosynthesis, a dynamic process essential for their development and survival [29,30]. This plant possesses essential genes that contribute to its adaptation and photosynthetic functions. The cp genome evolves rather slowly and has a highly stable structure. It is an excellent resource for studying plant phylogeny and evolutionary relationships. The cp genome, though, was derived from endosymbiotic bacteria and has retained a different identity in plant cells. The chloroplast genome provides a new target for genome editing in contemporary plant breeding programs [31]. The latest developments in high-throughput sequencing technologies and bioinformatic algorithms have greatly enhanced the efficiency, accuracy, and cost-effectiveness of cp genome assembly and phylogenomic research. Consequently, these developments have opened new avenues for utilizing cp genome variation in crop improvement, evolutionary biology, and molecular breeding [32]. The cp genomes of angiosperms commonly exhibit a large single-copy region (LSC), a short single-copy region (SSC), and two inverted repeat regions (IRa and IRb) [33]. This study presents the first fully assembled cp genome sequence of *C. bakerii* from Bangladesh. The findings will assist in advancing genetic markers for *C. bakerii* and are anticipated to offer a hypothetical foundation for genetic breeding, including the assessment of phylogenetic relationships.

## 2. Materials and Methods

### 2.1. Plant Materials, DNA Extraction

The *C. bakerii* leaf sample was collected from Chattogram, Bangladesh (22°28′10.87″ N; 91°46′49.82″ E) on 13 June 2023. The specimen was determined morphologically by Dr Shaikh Bokhtear Uddin, Professor, Department of Botany, University of Chittagong, Bangladesh, and deposited as a voucher specimen in the Chittagong University Herbarium. Genomic DNA was extracted from leaves with the DNeasy Plant Mini Kit, and the amount and percentage were evaluated using a Qubit fluorometer and agarose gel electrophoresis.

### 2.2. Sequencing and Assembly

Library preparation and sequencing were performed at BGI Genomics (Hong Kong) using the DNBseq platform. The raw reads were sorted out to remove low-quality sequences, adapter contamination, and other impurities using SOAPnuke v2.1.7, resulting in 64 GB of clean data comprising 150 bp paired-end reads. Genome assembly was conducted using GetOrganelle 1.7.7.1 and SPAdes 4.0 with Python 3.5.1 [34]. *Curcuma comosa*’ cp genome sequence (OK327014.1) was employed as the reference genome for the assembly of the *C. bakerii* genome. A circular contig including the whole cp genome sequence was produced for the species.

### 2.3. Gene Annotation

The entire cp of *C. bakerii* genomes was annotated using GeSeq [35,36] and amended by applying Sequin 15.5 (accessed on 25 February 2024). A circular visualisation of the cp genome was generated using OGDRAW 1.3.1. [37]. In the end, the cp genome sequencing results were submitted to GenBank. Finally, GenBank gave the following accession number: *C. bakerii* (Accession number: PV158104).

### 2.4. Codon Usage Bias and RNA Editing Site

MEGA12 was operated to determine the GC percentage of the screened coding sequences [38], while the first three spots of GC content three spots of the codons (GC1, GC2 and GC3, respectively) were determined with the CUSP applications within the EMBOSS explorer [39]. The proportion of the real codon usage of a codon in the gene under scrutiny to that projected for all codons in the gene is known as the relative synonymous codon usage (RSCU) value [40]. In addition, codon usage indices T3S, C3S, A3S, and G3S, which indicate nucleotide frequency at the third position of synonymous codons, as well as RSCU, effective number of codons (ENC) and Codon Adaptation Index (CAI), were analysed under CodonW v1.4.2 [41]. The pattern of synonymous codon usage is often evaluated to judge the influence of mutational bias and natural selection by using the reasoning that there is a relationship between the Parity Rule 2 plot and the distribution of the four bases in the third codon location of a gene. GC12 denotes the mean value of GC1 and GC2. A zero slope in the regression indicates that directional mutation pressure does not exist; for example, the codon usage pattern is due to selection pressure rather than mutational pressure. However, a slope of 1 indicates that mutational pressure affects GC12 and GC3 equally across codon positions. R software (version 3.6.3) was used [42]. Optimal codons were revealed using the ΔRSCU approach, where ΔRSCU denotes the diversity in RSCU values between highly and lowly expressed genes. A codon can be considered ideal when the ΔRSCU value is >0.08 and the resolved RSCU value in either the high- or low-expression dataset exceeds 1.

### 2.5. SSRs and Long Repeats Analyses

The local Microsatellite Identification web tools (MISA) technique was applied to predict the simple repetition sequence (SSR), with the maximum allowable counts for mononucleotide, dinucleotide, trinucleotide, tetranucleotide, pentanucleotide, and hexanucleotide repetitions established at 8, 5, 5, 4, 3, and 3, respectively. Furthermore, we used REPuter web-based software to analyze repetitive sequences, with complementary, forward, reverse, tandem, and palindromic repetitions. The minimum repeat length was set at 30 bp, and the maximum allowable edit distance was less than 3 bp [43].

### 2.6. IR Contraction and Expansion Analyses and Genomic Comparative and Nucleotide Diversity Analysis

We analyzed the cp genome boundaries of *C. bakerii* in comparison with nine other *Curcuma* species, focusing on the LSC, SSC, and IR regions, using annotated data generated through IRscope [44]. The Shuffle-LAGAN method in the mVISTA web application [42] was used to compare the nine *Curcuma* genomes with the *C. bakerii* genome as the reference. Results from the program showed overlapping gene names, which needed to be manually adjusted. Nucleotide diversity (Pi) for the cp genome was computed using DnaSP v5.1 using a sliding-window approach, employing a window length of 800 bp and a step size of 100 bp [45,46].

### 2.7. SNP-Rich Hypervariable Site Identification, Positive Selection Detection, Ka/Ks Calculation, and Phylogenetic Tree Construction

Scientists studied synonymous (Ks) and non-synonymous (Ka) substitution rates in *C. bakerii* to examine its molecular evolutionary dynamics relative to nine *Curcuma* species aligned. Using DnaSP v6, single-nucleotide polymorphisms (SNPs) were found from sequence alignments at variable sites, excluding alignment gaps [47,48]. Subsequently, the Ka/Ks ratios of each gene were calculated using the KaKs_Calculator v2.0 with the Yang–Nielsen method [49].

The cp genome sequences of 36 plant species were used for comparative analysis using the MAFFT-7.526 software [50]. A phylogenetic tree was created using the NJ and ML techniques in the MEGA 12 software [38], where the self-expansion evaluation of each parameter was set to 1000.

## 3. Results

### 3.1. Characteristics of C. bakerii

**Figure 1:**
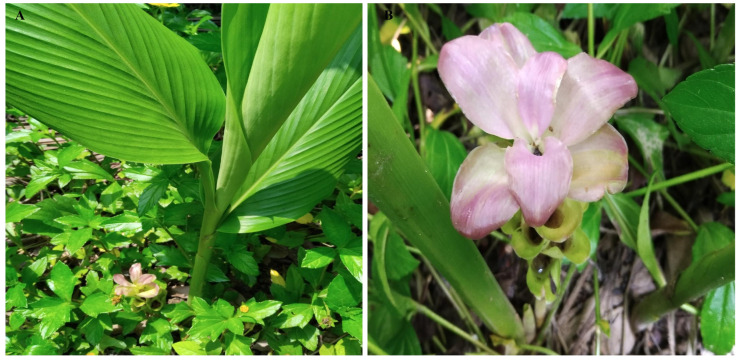
Morphological view of *C. bakerii.* (**A**) The plant, and (**B**) Flower.

**Figure 2:**
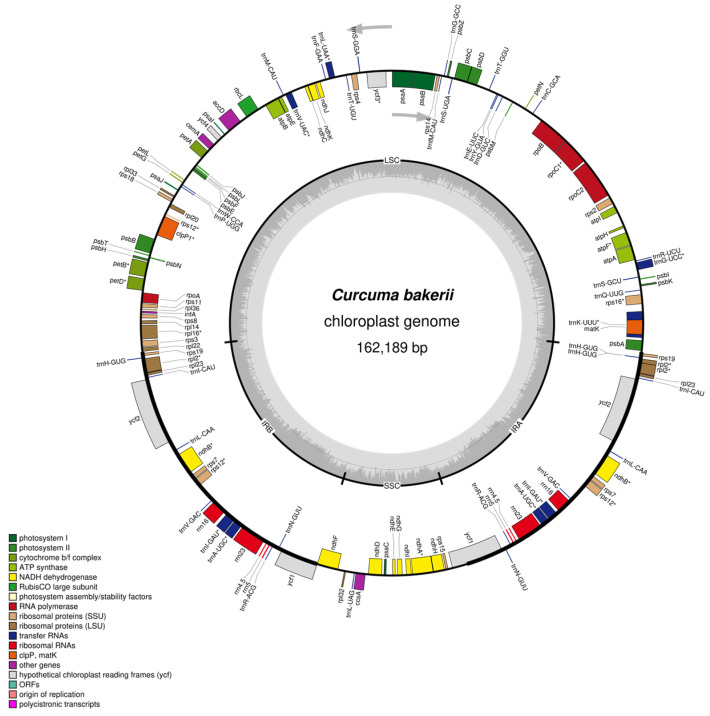
Circular representation of the *C. bakerii* cp genome. Genes located on the middle circle are recorded in the clockwise path, whereas individuals on the outward circle are recorded anti counterclockwise and innerward circle are clockwise direction. The dark grey ring illustrates the guanine–cytosine (GC) content, while the light grey ring designates the adenine–thymine (AT) content.

The Perennial, rhizomatous, herbaceous plants; stems generally absent or very short aboveground, leaves arise from pseudo stems formed by leaf sheaths, and the light purple colour of flowers (Figure 1). The cp genomes of *C. bakerii* were determined to be 162,189 bp in length and to exhibit a circular, quadripartite configuration (Figure 2). It had an LSC section of 87,024 bp, an SSC section of 15,665 bp, and two IR regions of 29,750 bp each (Figure 2). It comprised 135 genes overall, including 113 unique genes. These included 88 protein-coding genes (13 duplicated within the IR regions), eight rRNA genes (four unique), and 39 tRNA genes (30 unique) (Table S1). The percentage of GC content was found to be 36.18%. Furthermore, the IR areas had an elevated GC content, ranging from 41.13%. The LSC regions had a GC content of 33.97% across the genome. The SSC area showed the lowest GC content, averaging 29.66%.

### 3.2. Codon Usage Bias and RNA Editing Site

#### 3.2.1. RCSU and RFSC Analysis

The relative synonymous codon usage (RSCU) is retained to examine discrepancies in codon utilisation through genes. It measures how frequently a particular codon appears compared to how often it would be expected if all codons that are synonymous for the same amino acid were used evenly and without bias. The expected rate of a codon is determined by splitting the occurrence of its subsequent amino acid in the protein sequence by the synonymous codons that encode that amino acid. The RSCU rate of 1 suggests the absence of codon bias; values beyond 1 suggest a codon is used more often than anticipated, and values below 1 imply its comparative infrequency. The RSCU values in *C. bakerii* ranged from 0.41 to 2.00. All 31 codons with RSCU values over one were identified, indicating a preferential usage of these codons. Additionally, 34 codons that end with A or T (U) constituted 53.13% of all codons analyzed. Conversely, 30 codons showed RSCU < 1, including 30 codons ending with G or C nucleotides (46.88%), and three codons with RSCU = 1 exhibited no usage bias. Leucine had the greatest RSCU value among the amino acids, while methionine had the lowest. This means that codons tend to be used more often in sequences that are rich in leucine. We found 22 high-frequency codons that are regularly utilised in the cp genomes of Curcuma. These codons include UUU, UUA, UUG, CUU, AUU, GUU, GUA, UCU, UCA, CCA, ACA, GCU, GCA, UAU, CAU, CAA, AAA, GAU, GAA, UGU, and GGA. It showed the most common synonymous patterns in the plastid genomes of the genus (Table 1).

#### 3.2.2. Analysis of the Sources of Variation in Codon Usage Arrays

**Figure 3:**
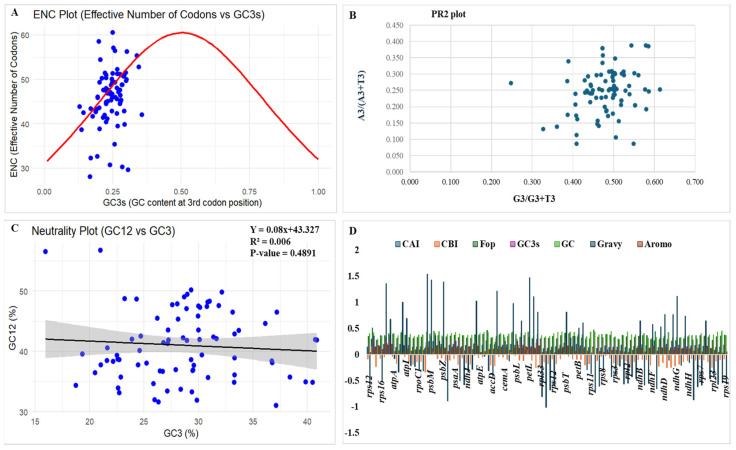
Codon usage bias analysis of the *C. bakerii* chloroplast genome included several graphical representations: (**A**) ENC plot, (**B**) PR2 plot, (**C**) Neutrality plot, and (**D**) codon usage indices, including the Codon Adaptation Index (CAI), Codon Bias Index (CBI), Frequency of Optimal Codons (Fop), GC content at the third codon position (GC3s), overall GC content (GC), Grand Average of Hydropathicity (GRAVY), and Aromaticity Index (Aromo), were analyzed to assess codon usage patterns.

The effective number of codons (ENC or Nc) is considered one of the most reliable indicators of overall synonymous codon usage bias. It can be assessed directly from codon usage data. To determine how codon usage changes across genes, ENC is calculated relative to factors such as GC content. The study of the ENC-GC3s plot (Figure 3A) was conducted to assess codon usage variation among chloroplast genes. The distribution of coding sequences (CDSs) of *C. bakerii* in the rectangular coordinate system showed a similar pattern across genes. Only a small number of CDSs deviated significantly, suggesting that mutational pressure had a minimal effect on the cp genome’s codon usage bias. According to the ENC value, the selected genes range from 28.1 to 60.57 (most between 40 and 55), indicating a moderate codon bias.

The study utilized the Parity Rule 2 (PR2) plot to evaluate the effects of mutational pressure and natural selection on the third codon position. The PR2 analysis distinguished between A/U (A3/[A3 + U3]) and G/C (G3/[G3 + C3]) biases. According to PR2 expectations, if the genome were influenced only by mutational bias, we should observe complementary base pairs (A = T and G = C) at similar frequencies. If this balance is disrupted, it means that selection on translational efficiency, asymmetric mutation, and other evolutionary forces are acting on synonymous sites. Points that cluster near the central position (0.5, 0.5) on the PR2 plot indicate that the use of complementary nucleotides is largely balanced, suggesting that codon usage is driven mainly by neutral mutational processes. To visualise these patterns, we generated a scatter plot of G3/(G3 + C3) vs. A3/(A3 + T3) (Figure 3B). The study revealed an A/T(U) bias of 0.491 and a G/C bias of 0.487, with overall AT and GC content of 63.05% and 36.95%, respectively. The distribution of coding sequences (CDSs) was asymmetrical about (A:U/T, G:C), suggesting that distortion due to mutation pressure and natural selection has been useful in modifying the codon usage in this organism.

To further test mutation pressure and selection and estimate their relative influence, neutrality plots of GC12 vs. GC3 were plotted (Figure 3C) and analysed by regression. The study showed that two variables combined to exert natural selection and mutational pressure on codon usage bias. There is a strong positive relationship between GC12 and GC3 levels, indicating that natural mutation pressure is the primary driver of codon usage preferences. The close relationship indicates that natural selection is the main reason. The regression curve failed to coincide with the diagonal graph, and the slope measures 0.4891. The correlation between GC3 and GC12 was weak, implying that the codon usage bias in *C. bakerii* is primarily shaped by natural selection rather than mutational pressure.

The cp genome of *C. bakerii* exhibited consistent codon use indices (Table S2), with CAI values reaching from 0.112 to 0.299, and most genes had values below 0.20. This means that most genes don’t adapt well to highly favoured codons. The CBI values were almost identical, with most genes showing negative or near-zero values, indicating modest codon bias. Frequency of Optimal Codons (Fop) values (0.16–0.51) showed that only a moderate number of optimum codons are employed, whereas most of the values were nearly 0.3. GC3s values (0.129–0.357) were usually low, as expected given the significant AT bias at synonymous third positions and the total genome GC content of 30–40%. Protein property indices further indicated that most plastid-encoded proteins are hydrophilic (exhibiting negative GRAVY scores) and possess low aromaticity (0.04–0.21). However, specific photosystem genes (*psbK*, *psbM*, *psbZ*, *petN*, *petL*, *petG*, *atphH*, *ndhC*, and *ndhG*) showed elevated GRAVY scores, highlighting the hydrophobicity of membrane-associated proteins (Figure 3D).

#### 3.2.3. Determination of Putative Optimal Codons

**Figure 4:**
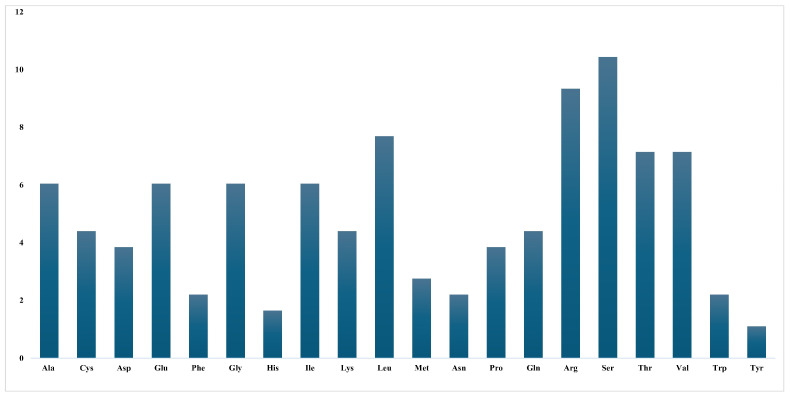
Relative synonymous codon usage (RSCU) values for 20 amino acids and stop codons across all protein-coding genes in the *C. bakerii* chloroplast genome.

The RSCU values of the 30 most frequently used codons in *C. bakerii* were analyzed, focusing on codons with RSCU values ≥1. Among them, 14 concluded with U, 13 with A, two with G, and just one with C, indicating a predilection for codons terminating in U. A qualified assessment was carried out to identify the five most optimal codons (defined by ΔRSCU > 0.08, including with RSCU > 1) across the studied species (Table S3). A two-way Chi-squared test was conducted to evaluate codon usage differences across genes, providing a basis for enhancing protein expression through codon optimisation strategies. Two proposed optimal codons were identified in the cp genome of *C. bakerii*. These included UCU, UCC, UCA for Ser, and AGA, AGG for Arg (Figure 4). The RSCU patterns in *C. bakerii* reveal a strong bias toward A/U-ending codons and identify only a few optimal codons (Ser: UCU/UCC/UCA; Arg: AGA/AGG), indicating that mutation pressure dominates codon usage with limited selective optimization.

#### 3.2.4. RNA Editing Sites

**Figure 5:**
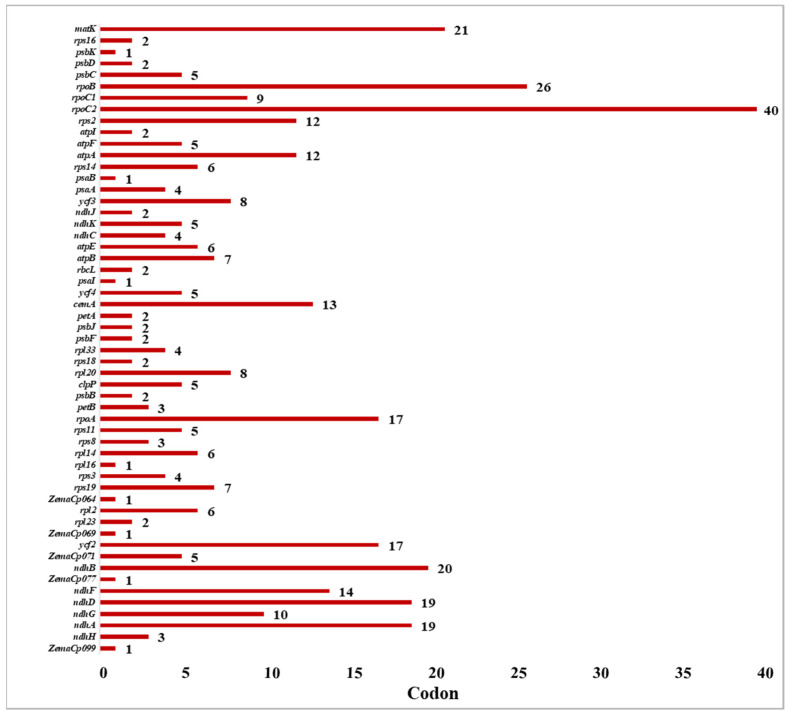
The predicted RNA editing site of the *C. bakerii* cp genome.

The PREPACT web database was used to identify C-to-U RNA editing locations in the *C. bakerii* cp genome. In total, 55 RNA editing locations were detected, allotted across 50 protein-coding and 5 non-coding genes (Table S4). Among these, the *rpoC2* gene had the most editing sites (40), followed by *rpoB* with 26. The matK gene had 21 sites, and the *ndhB* gene had 20 sites. On the other hand, the *ndhD* and *ndhA* genes each had 19 RNA editing sites (Figure 5). In addition, *rpoA* and *ycf2* both had 17 editing sites. In addition, some genes had 10–14 editing sites (*ndhG*, *cemA*, *atpA*, *rps2*, and *ndhF*), whereas the remaining genes had fewer than 10. The evidence indicates that RNA editing is not random, as it is marked by enrichment in transcription and energy metabolism genes. This suggests that it is vital for maintaining the functional proteins within the plastid.

### 3.3. SSRs and Long Repeats Analyses

**Figure 6:**
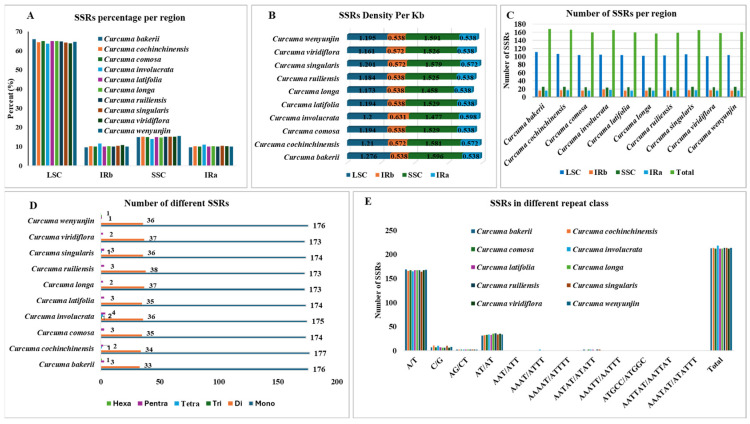
Comparison of simple sequence repeats (SSRs) among the cp genomes of ten *Curcuma* species. (**A**) Percentage allocation of SSRs across different genomic regions. (**B**) Frequency of SSRs density per kilobase (kb) in the LSC, SSC, and IR regions. (**C**) Number of SSR loci per region identified across species. (**D**) Number of different SSRs in the species (**E**) Frequency of SSRs categorised by repeat motif classes.

Simple sequence repeats or SSRs, otherwise known as microsatellites. They are small, positioned tandem DNA motifs that normally consist of one to six nucleotide pairs. Because there are repeats throughout the cp genome, they are useful molecular markers for species identification and genetic diversity assessment. Due to their high variability, SSRs, which are commonly used as molecular markers, have become a valuable tool in phylogenetic research, including population genetics. In this study, SSRs were predominantly located in the LSC and SSC sections of the chloroplast genome (Figure 6A), which represent the main areas of SSR distribution. The density of SSRs per Kb was the highest in the SSC region (1.477 to 1.596), and then the LSC region (1.2 to 1.276), whereas only a small number of densities were found within the two IR regions (0.538 to 0.572) (Figure 6B). There are 168 loci with SSRs in *C. bakerii*, including single hexanucleotides, three pentanucleotides, 33 dinucleotides, and 176 mononucleotides. Among 10 *Curcuma* species, having total loci counts between 157 (*C. longa*) to 168 (*C. bakerii*). The LSC region (101–111) had the most loci (63.92–66%), followed by the SSC (23–25), which had almost 15% and the remaining IRs (16–19), which had copies of the same genes. There were 212–218 SSRs in each species, whereas *C. bakerii* had 213 SSRs (Figure 6C). Mononucleotide repeats represented the most abundant class of SSRs, with their numbers ranging from 173 to 176. These were followed by dinucleotide repeats (33–38). Tetranucleotide SSRs were few, occurring in only three of the ten species (0–2 repeats), while trinucleotide SSRs were detected in just one species (0–1 repeat). Pentanucleotide repeats ranged from 1 to 4, and hexanucleotide SSRs were the least common, found in two species with 0–1 repeat each (Figure 6D). Among them, *C. bakerii* has one exceptional repeat AATTAT/AATTAT. Mononucleotide SSRs were the most common type of repeat, accounting for 80.84–82.71% of the total. Among the ten sequenced chloroplast genomes, *C. cochinchinensis* had the highest proportion (82.71%), followed by *C. bakerii* (82.63%). The AT/AT repeat type represented 15.49–17.75% of the total, while other repeat types contributed less than 2.25–3.5% (Figure 6E).

**Figure 7:**
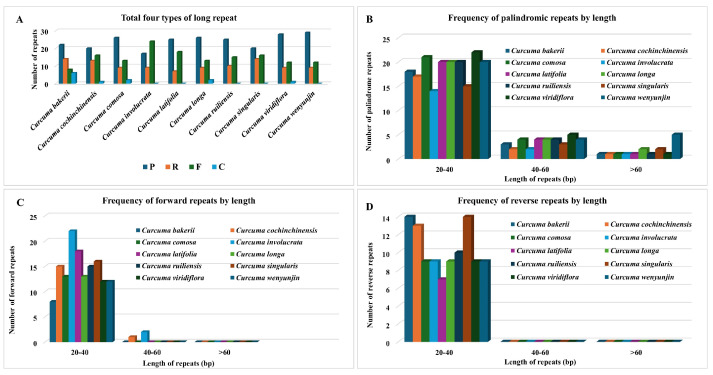
Analysis of long repeat sequences in the cp genomes of ten *Curcuma* plants. (**A**) Distribution of four repeat types: P (palindromic), R (reverse), F (forward), and C (complement). (**B**) Frequency of palindromic repeats by length. (**C**) Frequency of forward repeats by length. (**D**) Frequency of reverse repeats by length.

Long repeats (more than 30 bp) may help reconfigure the cp genome and promote genetic variety in populations, an area of genomics that has been receiving considerable attention. In this research, we also studied the repeated sequences of ten *Curcuma* cp genomes. Notably, the *C. bakerii* cp genome contained 22 palindromic repeats (8 forward, 14 reverse, and 6 complementary). Moreover, the number of these repeat types varied among *Curcuma* species, with palindromic repeats ranging from 17 to 29, and noticeable differences were observed in reverse repeats and abundance (7 to 14). In contrast, forward repeats (8 to 24) and complement repeats are less abundant (0 to 6) (Figure 7). Overall, the SSR and long-repeat patterns across the Curcuma plastomes reveal a strong AT-rich, LSC/SSC-biased distribution, providing a useful reservoir of variable markers for species identification and population-level phylogenetic studies.

### 3.4. IR Contraction and Expansion Analyses

**Figure 8:**
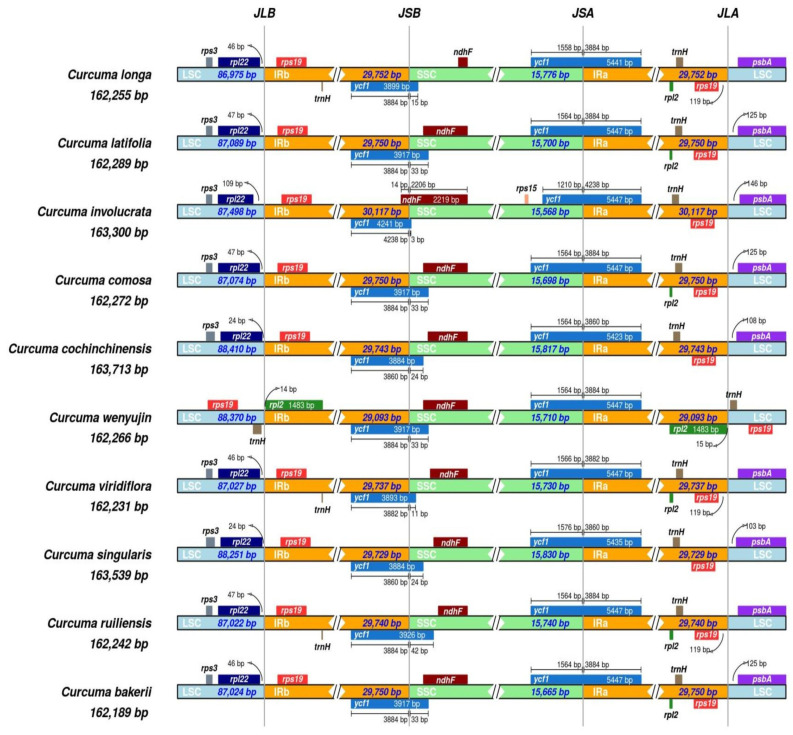
Comparison of junction boundaries among the LSC, SSC, and IR regions in the cp genomes of nine *Curcuma* species and *C. bakerii*. Distinctly coloured blocks represent complete or partial genes located adjacent to the junctions.

The research included the study of LSC/IR/SSC lines across the ten *Curcuma* species. The hypothesised shrinking and expansion of the IR borders may explain the variation in chloroplast genome size at the genus level. The IR region length across the ten *Curcuma* cp genomes exhibited only minor variation, ranging from 29,093 bp to 29,752 bp. In all genomes, the *rpl22* and *rps19* genes were positioned near the LSC/IRb junctions. The distance between *rpl22* and the LSC/IRb boundary ranged from 24 to 109 bp, whereas the distance between *rps19* and the same junction varied from 15 to 125 bp (Figure 8).

All ten *Curcuma* species contained the *ycf1* and *ndhF* genes near the boundaries of the IRb/SSC domains. The IRb/SSC borders among taxa were all situated at the end of *ycf1*. Moreover, *ycf1* expanded into the SSC regions across all species. These were *C. bakerii*, *C. latifolia*, *C. wenyujin*, and *C. comosa* for 33 bp, and 24 bp expanded by *C. singularis* and *C. cochinchinensis*, whereas the remaining species were 3 bp to 42 bp prolonged. The size of the *ycf1* gene within the IRa section varied among the ten *Curcuma* taxa, ranging from 1210 bp to 1576 bp. The *rps19* and *psbA* genes were positioned near the IRa/LSC junctions in all nine *Curcuma* species, except in *C. wenyujin*, where the gap between *rps19* and the IRa/LSC boundary ranged from 15 bp to 119 bp (Figure 8). A gap of 103–146 bp was identified between the *psbA* gene and the IRa/LSC boundary across all *Curcuma* species. Hence, the limited variation in IR length and the consistent placement of boundary-associated genes suggest that IR contraction and expansion events are minimal in *Curcuma*, with modest expansions of *ycf1* accounting for much of the plastome size variation across species.

### 3.5. Comparative Genomic and Nucleotide Diversity Analyses

**Figure 9:**
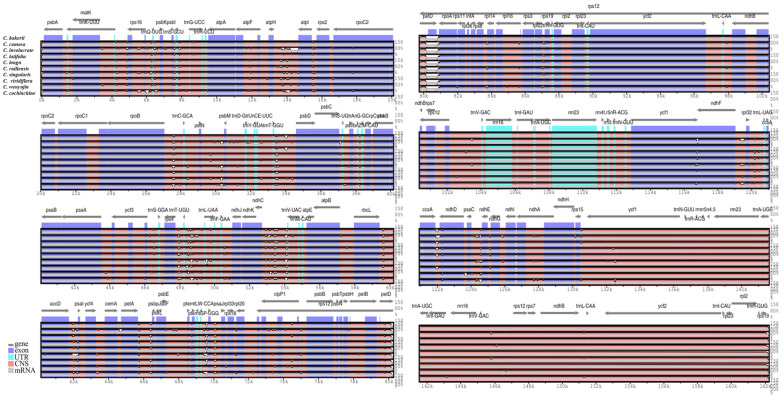
Comparative analysis of sequence identity among ten *Curcuma* chloroplast genomes, using the *C. bakerii* genome as the reference (upper plot). The mVISTA program was employed to visualise percentage sequence identity. Grey arrows with thick black lines denote gene orientation. Purple bars indicate exons, sky-blue bars represent untranslated regions (UTRs), red bars correspond to non-coding sequences (CNS), grey bars denote mRNA, and white areas mark regions of variation among the genomes. The horizontal axis shows chloroplast genome coordinates, while the vertical axis displays sequence identity values ranging from 50% to 100%. The ten cp genomes analysed in this study are highlighted in bold.

A comparative analysis of numerous alignments of ten *Curcuma* chloroplast genomes was organised using mVISTA, with the annotated *C. bakerii* genome sequence as the reference (Figure 9). The study demonstrated that the LSC and SSC regions showed higher levels of variation, whereas the two IR regions remained highly conserved across the cp genomes. Furthermore, the non-coding regions exhibited higher nucleotide divergence than the coding regions. The non-coding regions, strongly divergent regions were *trnL-UAA*, *trnF-GAA- trnV-UAC*, *trnM-CAU*, *trnC-GCA- psbM*, *trnD-GUC- trnT-GGU*, *trnS-GGA- trnL-UAA*, *trnV-UAC*, *trnV-GAC*, *trnL-UAG -trnH-GUG* and coding regions were *psbM*, *ndhC*, *rbcL-accD*, *ndhC*, *accD-psaI*, *atph-atpI*, *rps16- psbK*, *petA-psbE*, *petD-rpoA*, *psbE-rps18*, *ycf1*, *rpl32*, *ccsA-ndhD*, *pasC-ndhE*, *rps19* (Figure 9).

**Figure 10:**
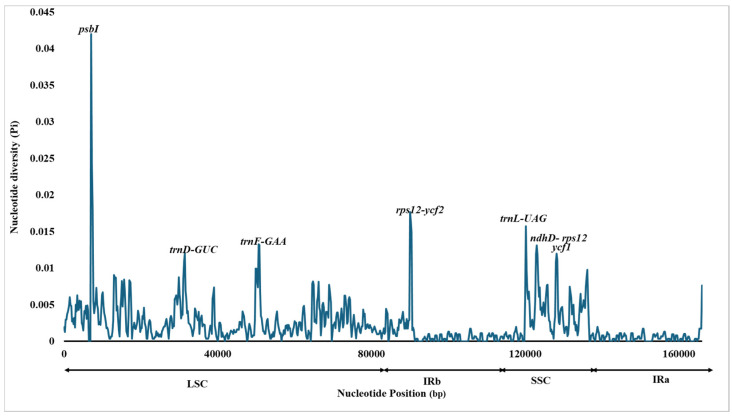
Sliding window analysis of ten *Curcuma* species based on their *C. bakerii* chloroplast genomes. The Y-axis represents the nucleotide diversity (Pi) values for each window, while the X-axis indicates the consistent genomic positions.

Additionally, sequence divergence hotspots were identified by calculating nucleotide diversity (Pi) within 800 bp windows (Table S4). The results showed that the nucleotide diversity (Pi) across the chloroplast genomes of Curcuma species ranged from 0 to 0.041. Seven highly variable areas (Pi > 0.01) were detected: *psbI*, *trnD-GUC*, *trnF-GAA*, *rps12-ycf2*, *trnL-UAG*, *ndhD-rps12*, *and ycf1*. Among these, in the LSC region, three hotspots, psbI, *trnD-GUC*, and *trnF-GAA*, were identified, whereas in one region, *rps12-ycf2* was found in IRb. The three regions, *trnl-UAG*, *ndhD-rps12*, and *ycf1*, were in the SSC region (Figure 10). Therefore, the comparative patterns indicate that while the *Curcuma* plastomes are broadly conserved, a small number of highly variable regions distributed across the LSC, SSC, and IR boundary areas offer valuable markers for distinguishing species and for interpreting evolutionary divergence within the genus.

### 3.6. Identification of SNPs with Hypervariable Regions and Favourable Selection Analysis/Synonymous and Non-Synonymous Substitution Rates

#### 3.6.1. Identification of SNPs with Hypervariable Regions

**Figure 11:**
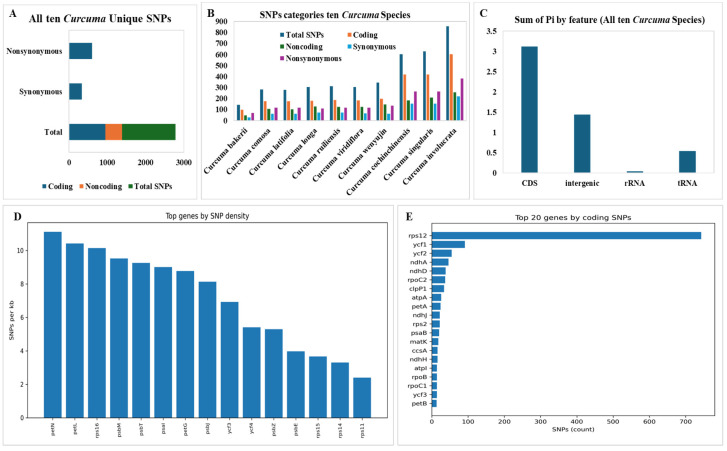
SNPs with hypervariable regions of *C. bakerii* and nine other *Curcuma* species. (**A**) Unique Synonymous and Nonsynonymous SNPs, (**B**) Total SNPs (Coding, Noncoding, Synonymous, Nonsynonymous), (**C**) Sum of Pi by feature, (**D**) Top genes by SNPs density, (**E**) Top 20 genes by coding SNPs.

We identified a total of 1391 unique SNPs, coding 947 SNPs (68.08%) (Synonymous 342 SNPs as 36.11%) (Nonsynonymous 605 as 63.78%) and noncoding 444 SNPs (31.91%) in ten Curcuma species that were used as the *C. bakerii* reference genome (Figure 11A). A total of 143 SNPs (*C. bakerii*) to 857 SNPs (*C. involucrate*) were detected in those ten *Curcuma* species. Although the range of coding SNPs 97 (*C. bakerii*) to 602 (*C. involucrata*) typically makes up about 60–70% of total SNPs, the remaining were noncoding. Protein function is maintained by SNPs that are in the range Synonymous (30–218) and Nonsynonymous SNPs (67–383) change amino acids and show how much potential selective pressure is acting on them (Figure 11B). The study of SNP density found that the genes *petN* (11.0 SNPs/kb), *petL* (10.4 SNPs/kb), and *rps16* (10.1 SNPs/kb) were the most different between *Curcuma* cp genomes. Photosynthesis and ribosome processes utilise these genes frequently, so they may be sites in the plastome where changes occur often. Ribosomal genes, such as *rps11* and *rps14*, have the fewest SNPs (<3 SNPs/kb), indicating that their roles have remained unchanged (Figure 11C). The cp genomes of *Curcuma* species are conserved, although the increasing number of SNPs in *C. cochinchinensis*, *C. singularis*, and *C. involucrata* suggests more evolutionary variability. Nonsynonymous SNPs in chloroplast protein-coding genes are common across all species, suggesting adaptive or lineage-specific evolution. The outline supports the typical evolution of chloroplast genomes, in which protein-coding (CDS) regions exhibited the highest nucleotide diversity (3.12) and intergenic regions (1.44) evolved more rapidly. In contrast, structural RNA (0.04) genes underwent stringent purifying selection to maintain their functional integrity (Figure 11D). The distribution of SNPs across chloroplast genes showed that *rps12* had the highest number of variable sites (743 SNPs), followed by *ycf1* and *ycf2*. The very high diversity in *rps12* is probably due to its trans-spliced gene structure and the high mutation density seen in many *Curcuma* species.

In general, the variation showed a balance between strong selection pressures on key genes and weaker selection pressures in areas that are not necessary or that do not control things.

#### 3.6.2. Selection Pressure Analysis

**Figure 12:**
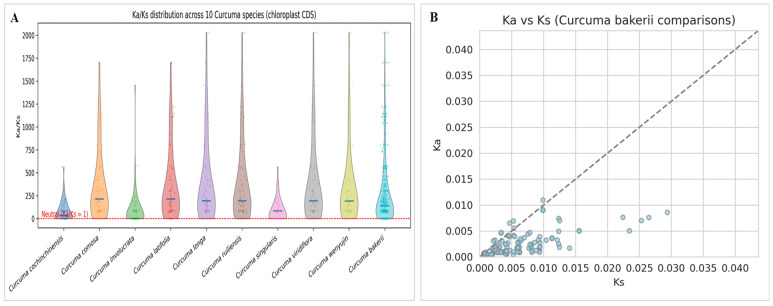
Distribution of synonymous (Syn) and nonsynonymous (Nonsyn) SNPs across selection pressure of the nine *Curcuma* species, including *C. bakerii*, which was the reference chloroplast genome. (**A**) Ka/Ks distribution of ten *Curcuma* species (CDS), (**B**) Ka/Ks of *C. bakerii* comparison.

Evolutionary pressure is another name for selection pressure. There are three main types of selection pressure: positive, neutral, and purifying. In genetics, Ka/Ks, also written as dN/dS, is the ratio of heterozygous substitutions (Ka) to homozygous substitutions (Ks). These numbers are called Ka and Ks. Ka represents the quantity of SNPs with non-synonymous substitutions and Ks is the calculate of SNPs with homozygous substitutions. By looking at the Ka/Ks ratio, we can tell if the selection pressure changes genes that code for proteins. When the Ka/Ks ratio exceeds 1, a positive selection effect is observed. When it equals 1, a negative selection effect is observed, indicating that they are evolving neutrally or nearly neutrally, with an equal chance of having synonymous or nonsynonymous substitutions. When it is less than 1, it designates purifying selection. According to Ka/Ks distribution across 10 *Curcuma* cp genomes, the maximum number of protein-coding genes is under strong purifying selection to stabilize chloroplast-encoded proteins. This is because Ka/Ks < 1. The median Ka/Ks values differed marginally among species, with *C. comosa*, *C. latifolia*, *C. longa*, *C. ruiliensis*, and *C. wenyujin* exhibiting comparatively elevated Ka/Ks ratios in contrast to *C. cochinchinensis* and *C. singularis*. It suggests that selection or adaptive divergence is relaxing moderately in certain lineages. The broader ranges observed in species such as *C. comosa* and *C. longa* might reflect greater genetic diversity than those of other species. Maybe it is due to selection pressures between photosynthetic and ribosomal genes. In contrast, *C. bakerii* showed a narrow Ka/Ks distribution, with values near zero (Figure 12A). This is the line with excellent functional conservation and few nonsynonymous alterations. The findings indicate that chloroplast genes in *Curcuma* are mostly conserved. However, some lineages may have undergone species-specific adaptive evolution, particularly in genes associated with photosynthesis and energy metabolism. We compared Ka and Ks in the coding sequences of *Curcuma* chloroplasts and *C. bakerii*. We found low change rates (Ka and Ks < 0.04) in the pairwise Ka–Ks associations, and they are very similar (Figure 12B). Most of the points on the Ka–Ks plots between *C. bakerii* and other *Curcuma* species were below the neutral line (Ka = Ks). The information showed that the chloroplast protein-coding areas are mostly the same within the genus and only slightly different between species.

### 3.7. Phylogenetic Analysis

**Figure 13:**
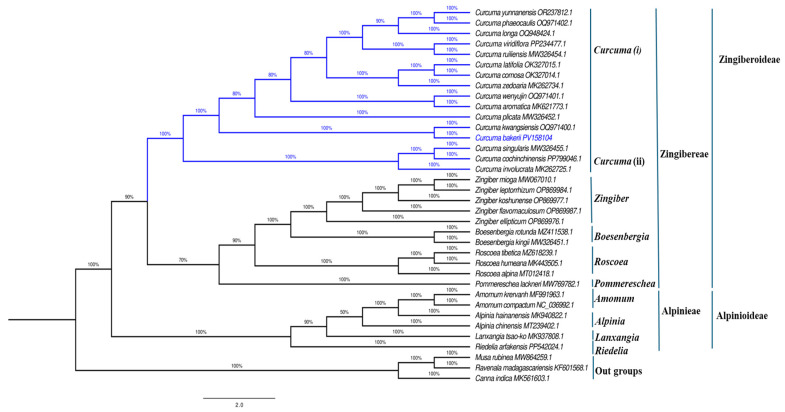
A maximum-likelihood (ML) phylogenetic tree was constructed from cp genome sequences of 36 species. The numerical values displayed beside each branch indicate the bootstrap support scores. The blue color indicate curcuma.

To examine the phylogenetic locations of *C. bakerii* and its relationships within other species of the Zingiberaceae family. The order Zingiberales was investigated using maximum likelihood phylogenetic analyses of cp genomes from 36 species within the Zingiberaceae family. Notably, only three species outside of the family (Musaceae, Strelitziaceae, Cannaceae) were found to belong to Zingiberales. In this analysis, two subfamilies, Zingiberoideae and Alpinioideae, were separated into two tribes with 100% bootstrap support (BS). One tribe, Zingibereae, contained five genera (*Curcuma*, *Zingiber*, *Boesenbergia*, *Roscoea*, and *Pommereschea*). The second group, tribe Alpinieae, involved four genera (Amomum, Alpinia, Lanxangia, Riedelia) (BS = 100%), and *C. bakerii* was placed in the Zingibereae tribe. The whole cp genome sequencing of *C. bakerii* will serve as a valuable resource for the conservation genetics of this plant and for phylogenetic investigations within Zingiberaceae. The phylogenetic reconstruction also showed that *C. bakerii* clustered monophyletically with other *Curcuma* species, with high support. Likewise, they and other Zingibereae are stable within the gene tree. The high bootstrap values across species support complete cp genomes, providing ample phylogenetic signal to resolve intergenic and intragenic relationships within Zingiberaceae. This study provides a further valuable case demonstrating the usefulness of plastome-scale datasets for enhancing taxonomic resolution and tracing diversification patterns in this clade of Zingiberoideae, noting the clear separation of genus *Curcuma* from closely allied genera *Zingiber*, *Boesenbergia*, and *Roscoea*. This study further established the importance of the created *C. bakerii* plastome as a reference for future studies, including comparative, evolutionary, and conservation studies in the family, see Figure 13.

## 4. Discussion

The cp genome of *C. bakerii* was newly characterized in this analysis. The entire length is 162,189 base pairs, with a GC content of 37.86%. The genome exhibits the typical quadripartite shape, and its gene composition, including all protein-coding genes, transfer RNAs, and ribosomal RNAs, shows a high degree of similarity to other members of the Zingiberoideae subfamily [51,52]. The codon use bias indicates origin, mutation trends, and evolution of taxa or genes, exhibiting variances across the genomes of various creatures [53].

There were 21 identical codons (RSCU < 1), and three of the codons are common (RSCU > 1). These three codons show no bias in this species. Codon usage bias indicates the source, mutation, and evolution of species or genes and exhibits variation across the genomes of various plants [54]. Our analysis has shown that more codons (RSCU > 1) exhibit codon use bias in the cp genome of *Curcuma* species [26]. Codon preferences in cp genomes are important for gene expression efficiency. It also indicates mutation patterns that happened during evolution [55]. The effective number of codons (ENC) ranged from 28.1 to 60.57. Most of the genes were between 40 and 55. Thus, it shows a moderate bias in codon use. An analysis of the PR2 plot showed strong natural selection and high mutational pressure at the third codon position. The findings show that codon usage bias is more strongly influenced by natural selection than by mutational pressure. To understand codon usage bias across organisms, mutational pressure and selective forces must be balanced [56,57]. Most genes do not seem to adapt well to optimally favored codons. Most genes have negative or zero CBI values, indicating low codon bias. A relationship was observed between CAI and CBI in terms of the frequency of optimal codons and the base composition of codons. This means that a high GC base composition can significantly affect codon usage bias. Most plastid-encoded proteins are hydrophilic (with negative GRAVY scores) and exhibit low aromaticity (0.04–0.21). The observations indicate that the preferences in codon usage among various plant species are strongly determined by the hydrophobicity and aromaticity [58,59]. The RSCU analysis of *C. bakerii* identifies 30 preferred codons, including five optimal codons, and 55 RNA editing sites. These data indicated that codon use in the cp genome is mainly governed by AT-rich mutational pressure, and selection constraints exert a stronger influence on photosynthesis-associated genes [42]. RNA editing in organelles of land plants is believed to have a monophyletic origin, affecting gene expression and functional traits [60]. *C. bakerii* was assessed to have 168 SSR loci, the highest number among the 10 *Curcuma* species studied, with a total SSR locus number ranging from 157 to 168, the species having 212–218 SSRs, where *C. bakerii* had 213. Most repeats were mononucleotide motifs in non-coding regions and contributed to the genome’s overall AT-richness. These new findings correlate with most reported angiosperm [61,62]. Curcuma has more palindromic (17–29) and reverse (7–14) repetitions than forward (8–24) and complement repeats. The SSRs and long repeats discovered in this study can be useful for molecular studies such as genetic diversity and relatedness, phylogenetics, species identification and evolution [63,64].

The primary reason for the variation observed in the plant cp genes is due to the contraction and extension of the IR region. The IR regions (IRa and IRb) are highly conserved regions of the cp genome [65]. On the other hand, the IR region is conserved across species within the same genus, such as *C. bakerii*. The differences were most evident in the arrangement and extension of genes such as *rps19* and *ndhF* at IR junctions, including LSC/IRb, IRb/SSC, and SSC/IRa, which changes in the structure of the cp genome, particularly in the IR region and arrangement of genes at IR borders, may be associated with speciation and biological evolution [66,67].

Molecular markers can be used to identify species by targeting hypervariable regions [68]. Nucleotide diversity is a measure of sequence divergence which provides insight into diversity within and between species [69]. The cp gene showing the greatest variation was *rps12*, followed by *ycf1* and *ycf2.* Several *Curcuma* species possess a trans-spliced gene structure and a high mutation density, which may contribute to the diversity of *the rps12 gene*. The SNP density analysis indicated that *petN*, *petL*, and *rps16* varied widely across the *Curcuma* cp genomes. They are associated with photosynthesis and ribosomes and may be important loci for plastome evolution. In addition, *rps11* and *rps14* have relatively few SNPs, which means that they are extremely functionally stable under purifying selection [18]. In addition to *rps11* and *rps14*, which have relatively few SNPs, they are extremely functionally stable under purifying selection. The higher variability observed in non-coding regions, particularly in intergenic spacer regions, for example, *psbI*, *trnD-GUC*, *trnF-GAA*, *rps12–ycf2*, *trnL-UAG*, *ndhD–rps12*, and *ycf1*, corroborates earlier studies in other angiosperms, and these regions are hotspots of sequence divergence and good candidates for molecular markers [70]. The sliding window analysis of average nucleotide diversity (Pi) gave values resembling those recorded in closely related genera suggesting that Pi analysis is reliable and can detect mutation hotspots useful for taxonomic differentiation [71].

The Ka/Ks distribution of 10 *Curcuma* cp genomes showed that most protein-coding genes were under strong purifying selection, thereby maintaining cp protein stability. These results reflect strong functional conservation of cp genes in *Curcuma*, with only limited non-synonymous changes. While most genes remain highly conserved, some lineages show signs of adaptive evolution, particularly in genes related to photosynthesis and energy metabolism. The Ka/Ks analysis supports this, as most points fell below the neutral line (Ka = Ks), indicating that purifying selection maintains the stability of cp protein-coding regions across. No non-synonymous variants are found in most cp genes and genic regions, indicating high selection pressure [72]. In the last 30 years, cp genomes in plants have been widely employed in evolutionary and phylogenetic studies due to their highly conserved sequences, simple and compact structure, and the rare incidence of horizontal gene transfer [73]. Recent progress in streptophyte phylogenomics, driven by extensive sequencing of chloroplast and nuclear genomes, has greatly improved our understanding of major plant lineages [74]. These advances underscore the importance of plastome-based analyses, such as those presented here for *Curcuma*, in clarifying evolutionary relationships and supporting more robust taxonomic interpretations.

Complete cp genomes provide enough informative locations for elucidating evolutionary connections among plants and have proven efficient in differentiating lower taxonomic groups [75]. *C. bakerii* was very closely related to the *C. kwangsiensis* of the *Curcuma* group and the position of the Zingibereae tribe and Zingiberoideae subfamily. The entire *C. bakerii* cp genome sequence serves as a valuable genetic resource for the conservation and genetic management of this species, for population genetics studies, and for phylogenetic analyses within the Zingiberaceae family. The placement of *C. bakerii* also contributes new comparative material for the genus, which remains underrepresented in plastome databases despite its high species richness [76]. Beyond phylogeny, the availability of the complete cp genome offers an important foundation for population genetics, species identification, conservation planning, and future comparative genomic work across Zingiberaceae [52]. As more *Curcuma* plastomes become available, these data will help refine our understanding of evolutionary divergence, lineage diversification, and biogeographic history within the family.

## 5. Conclusions

In conclusion, we described the entire cp genome sequencing and assessment of *C. bakerii*, an endemic plant of Bangladesh. According to our studies, this is the first report of the cp genome from *C. bakerii* in Bangladesh. Comparative cp genome detected 13 high codons and eight putative optimal codons. Overall, 212–218 SSRs and long repeat sequences were recognized across the cp genomes of 10 *Curcuma* species. Seven divergent areas (*psbI*, *trnD-GUC*, *trnF-GAA*, *rps12-ycf2*, *trnL-UAG*, *ndhD-rps12*, and *ycf1*) were identified. These markers may prove useful for future detection of *Curcuma* species and phylogenetic analyses within the genus. These results enhance understanding of genetic diversity within the *Curcuma* species, providing essential data for taxonomic and phylogenetic research. This is the first full cp genome sequencing of Bangladesh; therefore, it will be useful for future research. It is advisable to conduct further cp genome sequencing of several *Curcuma* species to improve comparative analysis, establish species-specific markers, and clarify taxonomic and phylogenetic ambiguities within the Zingiberaceae family.

## Figures and Tables

**Table 1:** RSCU and RFSC values of the codons in the chloroplast genome of *C. bakerii*.

Amino Acid	Codon	Count	RSCU	RSFC	Amino Acid	Codon	Count	RSCU	RSFC
Phe	UUU (F)	2414	1.24	0.62	Tyr	UAU (Y)	1720	1.37	0.68
	UUC (F)	1471	0.76	0.38		UAC (Y)	798	0.63	0.32
Leu	UUA (L)	1177	1.34	0.22	Stop	UAA (*)	1257	1.26	0.62
	UUG (L)	1087	1.24	0.21		UAG (*)	769	0.77	0.38
	CUU (L)	1068	1.22	0.20	His	CAU (H)	958	1.4	0.70
	CUC (L)	644	0.74	0.12		CAC (H)	415	0.6	0.30
	CUA (L)	815	0.93	0.16	Glu	CAA (Q)	1072	1.41	0.71
	CUG (L)	465	0.53	0.09		CAG (Q)	444	0.59	0.29
Iso	AUU (I)	1960	1.23	0.41	Asp	AAU (N)	1955	1.42	0.71
	AUC (I)	1067	0.67	0.22		AAC (N)	803	0.58	0.29
	AUA (I)	1757	1.1	0.37	Lys	AAA (K)	2304	1.37	0.69
Met	AUG (M)	944	1	1.00		AAG (K)	1049	0.63	0.31
Val	GUU (V)	788	1.29	0.32	Aspartic	GAU (D)	1127	1.45	0.72
	GUC (V)	469	0.77	0.19253		GAC (D)	431	0.55	0.28
	GUA (V)	776	1.27	0.31856	Glutamic	GAA (E)	1411	1.41	0.70
	GUG (V)	403	0.66	0.16544		GAG (E)	594	0.59	0.30
Ser	UCU (S)	1214	1.47	0.32469	Cys	UGU (C)	751	1.25	0.62
	UCC (S)	936	1.13	0.25033		UGC (C)	453	0.75	0.38
	UCA (S)	1001	1.21	0.26772	Stop	UGA (*)	965	0.97	1.00
	UCG (S)	588	0.71	0.15726	Try	UGG (W)	690	1	1.00
Pro	CCU (P)	684	1.16	0.29008	Arg	CGU (R)	399	0.72	0.25
	CCC (P)	561	0.95	0.23791		CGC (R)	227	0.41	0.14
	CCA (P)	735	1.25	0.3117		CGA (R)	597	1.07	0.37
	CCG (P)	378	0.64	0.16031		CGG (R)	373	0.67	0.23
Thr	ACU (T)	721	1.19	0.29867	Ser	AGU (S)	747	0.9	0.61
	ACC (T)	601	1	0.24896		AGC (S)	471	0.57	0.39
	ACA (T)	728	1.21	0.30157	Arg	AGA (R)	1111	2	0.64
	ACG (T)	364	0.6	0.15079		AGG (R)	628	1.13	0.36
Ala	GCU (A)	505	1.32	0.33007	Gly	GGU (G)	591	1.08	0.27
	GCC (A)	325	0.85	0.21242		GGC (G)	316	0.58	0.14
	GCA (A)	477	1.25	0.31176		GGA (G)	805	1.46	0.37
	GCG (A)	223	0.58	0.14575		GGG (G)	486	0.88	0.22

Note: “*” indicate stop codon.

## Data Availability

The chloroplast genome sequence obtained in this work has been submitted to GenBank (accession number: PV158104). The data will become publicly accessible after the manuscript is published.

## Supplementary Materials

The following supporting information can be downloaded at: https://www.mdpi.com/article/10.3390/genes16121460/s1.

## Abbreviations

AROMO	Aromaticity
CP	Chloroplast genome
CAI	Codon Adaptation Index
CBI	Codon Bias Index
ENC	Effective Number of Codons
FOP	Frequency of Optimal Codons
GRAVY	Grand Average of Hydropathicity
IR	Inverted Repeat
LSR	Large Single Region
LSC	Large Single-Copy Region
ML	Maximum Likelihood
MISA	Microsatellite Identification Tool
PR2	Parity Rule 2 Plot
RSCU	Relative Synonymous Codon Usage
RFSC	Relative Frequency of Synonymous Codons
SSC	Small Single-Copy Region
SSR	Simple Sequence Repeat
SNP	Single Nucleotide Polymorphism
