# Supplementary material for: The Complete Chloroplast Genome of Curcuma bakerii, an Endemic Medicinal Plant of Bangladesh: Insights into Genome Structure, Comparative Genomics, and Phylogenetic Relationships
Source: Genes (Basel). 2025 Dec 7;16(12):1460. doi: 10.3390/genes16121460 (PMC12732962; doi:10.3390/genes16121460)
Supplement: Supplementary file 1 [file genes-16-01460-s001.zip › Table S1.pdf]

Table S1: Gene composition of *Curcuma bakerii* chloroplast genome

Category for genes	Groups of genes	Name of genes
**Photosynthesis-related genes**	Large unit of Rubisco	*rbcL*
	Photosystem I	*psaA, psaB, psaC, psal, psaJ*
	Assembly/stability of photosystem I	*ycf3 ,ycf4*
	Photosystem II	*psbA, psbB, psbC, psbD, psbE, psbF, psbH, psbI, psbJ, psbK, psbL, psbM, psbN, psbT, psbZ*
	ATP synthase	*atpA atpB, atpE,atpF, atpH, atpl*
	Cytochrome b/f complex	*petA, petB, petD, petG, petL, petN*
	Cytochrome c synthesis	*ccsA*
	NADPH dehydrogenase	*ndhA, ndhB(x2), ndhC, ndhD, ndhE, ndhF, ndhG, ndhH, ndhl, ndhJ, ndhK*
**Transcription and translation-related genes**	Transcription	*rpoA, rpoB, rpoC1, rpoC2*
	Ribosomal proteins	*rps2, rps3, rps4, rps7(x2), rps8, rps11, rps12(x2), rps14, rps15, rps16, rps18, rps19(x2)*
	Translation initiation factor	*infA*
**RNA genes**	Ribosomal RNA	*rrn4.5(x2), rrn5(x2), rrn16(x2), rrn23(x2)*
	Large ribosomal subunit	*rpl2(x2), rpl14, rpl16, rpl20, rpl22, rpl23(x2), rpl32, rpl33, rpl36*
	Transfer RNA	*trnA-UGC(x2), trnC-GCA, trnD-GUC, trnE-UUC, trnF-GAA, trnfM-CAU, trnG- UCC1, trnG-GCC, trnH-GUG(×2), trnl-CAU(×2), trnl-GAU(×2), trnK-UUU, trnL-CAA(×2), trnL-UAA, trnL-UAG, trnM-CAU, trnN-GUU(×2), trnP-UGG, trnQ-UUG, trnR-ACG(×2), trnR-UCU, trnS-GCU, trnS-GGA, trnS-UGA, trnT-GGU, trnT-UGU, trnV-GAC(×2), trnV-UAC, trnW-CCA, trnY-GUA*
**Other genes**	RNA processing	*matK*
	Carbon metabolism	*cemA*
	Fatty acid synthesis	*accD*
	Proteolysis	*clpP1*
**Genes of unknown function**	Conserved reading frame	*ycf1(x2), ycf2(x2)*
