# Supplementary material for: The Complete Chloroplast Genome of Curcuma bakerii, an Endemic Medicinal Plant of Bangladesh: Insights into Genome Structure, Comparative Genomics, and Phylogenetic Relationships
Source: Genes (Basel). 2025 Dec 7;16(12):1460. doi: 10.3390/genes16121460 (PMC12732962; doi:10.3390/genes16121460)
Supplement: Supplementary file 1 [file genes-16-01460-s001.zip › Table S2.pdf]

Table S2: Codon usage bias indices of protein-coding genes in the *C. bakerii* chloroplast genome.

Title	CAI	CBI	Fop	ENc	GC3s	GC	Gravy	Aromo
rps12	0.145	-0.08	0.352	41.97	0.221	0.407	-0.59594	0.04065
psbA	0.299	0.159	0.511	44.84	0.278	0.417	0.358357	0.135977
matK	0.153	-0.227	0.276	50.22	0.211	0.296	0.002913	0.178641
rps16	0.164	-0.078	0.366	43.37	0.171	0.361	-0.52651	0.096386
psbK	0.198	-0.045	0.362	49.34	0.259	0.344	1.362295	0.213115
psbI	0.186	0.016	0.4	30.23	0.286	0.37	0.675	0.194444
atpA	0.197	-0.036	0.394	45.99	0.249	0.406	-0.07712	0.059172
atpF	0.136	-0.168	0.313	39.45	0.268	0.362	-0.32935	0.076087
atpH	0.199	0.033	0.418	49.34	0.203	0.453	1.002469	0.049383
atpI	0.175	-0.04	0.37	44.87	0.227	0.368	0.689879	0.117409
rps2	0.172	-0.144	0.33	47.6	0.277	0.388	-0.21356	0.067797
rpoC2	0.149	-0.178	0.31	46.88	0.23	0.349	-0.2272	0.092364
rpoC1	0.147	-0.132	0.33	47.13	0.229	0.374	-0.30526	0.092105
rpoB	0.151	-0.131	0.338	46.72	0.249	0.379	-0.3097	0.082255
petN	0.119	-0.333	0.16	30.7	0.24	0.437	1.544827	0.137931
psbM	0.249	0.012	0.394	0	0.273	0.343	1.429412	0.117647
psbD	0.253	0.056	0.45	41.11	0.23	0.41	0.362323	0.169972
psbC	0.179	-0.072	0.366	44.53	0.277	0.442	0.262039	0.136659
psbZ	0.201	-0.058	0.356	38.63	0.136	0.317	1.387097	0.112903
rps14	0.131	-0.115	0.344	45.32	0.281	0.39	-0.889	0.06
psaB	0.183	-0.104	0.357	48.01	0.266	0.409	0.120981	0.148501
psaA	0.201	-0.073	0.372	48.73	0.286	0.427	0.262933	0.130667
ycf3	0.158	-0.188	0.327	50.71	0.29	0.397	-0.50298	0.130952
rps4	0.155	-0.057	0.369	46.63	0.242	0.365	-0.57861	0.074627
ndhJ	0.177	-0.156	0.32	50.08	0.247	0.375	-0.33522	0.150943
ndhK	0.156	-0.178	0.311	49.24	0.249	0.371	-0.2836	0.096
ndhC	0.209	-0.013	0.384	56.43	0.259	0.383	1.026667	0.2
atpE	0.163	-0.06	0.374	45.96	0.26	0.405	0.040741	0.037037
atpB	0.186	-0.042	0.385	47.16	0.271	0.423	-0.03293	0.060606
rbcL	0.269	0.067	0.463	46.7	0.246	0.425	-0.31333	0.097917
accD	0.201	-0.197	0.322	42.63	0.189	0.329	-0.41726	0.107843
psaI	0.188	-0.214	0.235	39.82	0.294	0.352	1.216667	0.111111
ycf4	0.158	-0.04	0.382	49.96	0.301	0.404	0.135326	0.146739
cemA	0.184	-0.046	0.377	47.06	0.242	0.325	0.262882	0.148472
petA	0.189	-0.091	0.352	52.23	0.267	0.395	-0.12281	0.0875
psbJ	0.195	0.043	0.421	0	0.289	0.433	0.98	0.125
psbL	0.183	-0.015	0.389	51.37	0.222	0.298	0.257895	0.184211
psbF	0.123	-0.108	0.333	51.31	0.25	0.419	0.646154	0.128205
psbE	0.185	-0.111	0.363	55.32	0.338	0.418	0.075903	0.156627
petL	0.14	0.085	0.433	58.5	0.2	0.333	1.470968	0.129032
petG	0.15	-0.103	0.306	32.64	0.194	0.342	1.113513	0.135135
psaJ	0.116	-0.253	0.225	49.66	0.3	0.397	0.816667	0.166667
rpl33	0.17	-0.211	0.308	42.86	0.292	0.369	-0.80455	0.045455

rps18	0.112	-0.141	0.321	35.35	0.257	0.342	-1.01261	0.063063
rpl20	0.121	-0.173	0.314	51.18	0.277	0.35	-0.67254	0.084507
rps12	0.145	-0.08	0.352	41.97	0.221	0.407	-0.59594	0.04065
clpP1	0.151	-0.174	0.307	56.28	0.302	0.423	0.07561	0.112195
psbB	0.18	-0.083	0.366	45.59	0.266	0.436	0.117716	0.145669
psbT	0.219	0.036	0.412	54.41	0.206	0.314	0.814286	0.171429
psbN	0.168	-0.031	0.405	42.05	0.357	0.434	0.323256	0.139535
psbH	0.164	-0.082	0.353	42.34	0.265	0.425	0.346575	0.068493
petB	0.198	-0.093	0.345	41.43	0.215	0.392	0.506512	0.134884
petD	0.174	-0.088	0.322	47.6	0.217	0.381	0.605	0.10625
rpoA	0.146	-0.197	0.296	50.33	0.227	0.343	-0.28397	0.072886
rps11	0.136	-0.171	0.308	42.48	0.143	0.435	-0.36232	0.028986
rpl36	0.144	0.12	0.472	29.65	0.306	0.432	-0.72703	0
infA	0.123	-0.111	0.333	51.21	0.267	0.377	-0.60649	0.064935
rps8	0.127	-0.033	0.391	38.82	0.203	0.331	-0.30606	0.045455
rpl14	0.169	-0.025	0.387	40.42	0.227	0.388	0.090164	0.040984
rpl16	0.135	-0.064	0.376	45.04	0.232	0.444	-0.42721	0.080882
rps3	0.146	-0.155	0.33	45.32	0.245	0.341	-0.38165	0.087156
rpl22	0.145	-0.196	0.298	43.89	0.129	0.292	-0.22016	0.069767
rps19	0.187	-0.084	0.375	44.47	0.227	0.366	-0.5587	0.054348
rpl2	0.134	-0.118	0.346	51.49	0.298	0.434	-0.54621	0.046931
rpl23	0.115	-0.241	0.276	57.09	0.253	0.38	-0.42473	0.086022
ycf2	0.159	-0.136	0.338	52.8	0.345	0.377	-0.46346	0.123843
ndhB	0.162	-0.094	0.349	46.43	0.279	0.375	0.644706	0.129412
rps7	0.189	-0.092	0.369	46.04	0.195	0.4	-0.58516	0.051613
ycf1	0.174	-0.1	0.365	47.62	0.234	0.311	-0.5449	0.123372
ndhF	0.133	-0.2	0.292	41.68	0.177	0.314	0.575068	0.170732
rpl32	0.264	0.014	0.444	28.1	0.167	0.327	-0.65439	0.070175
ccsA	0.139	-0.219	0.288	47.91	0.227	0.317	0.529787	0.151976
ndhD	0.131	-0.149	0.31	48.66	0.245	0.358	0.764882	0.141328
psaC	0.199	-0.246	0.289	60.57	0.25	0.424	-0.10864	0.074074
ndhE	0.13	-0.24	0.258	42.87	0.247	0.327	0.770297	0.089109
ndhG	0.143	-0.216	0.266	45.84	0.195	0.333	1.11875	0.119318
ndhI	0.187	-0.178	0.318	43.23	0.191	0.326	-0.20333	0.111111
ndhA	0.119	-0.137	0.304	43.58	0.201	0.357	0.736639	0.110193
ndhH	0.153	-0.116	0.339	50.98	0.227	0.374	-0.15038	0.117048
rps15	0.165	-0.075	0.364	32.31	0.17	0.281	-0.86222	0.066667
ycf1	0.175	-0.104	0.364	47.72	0.228	0.298	-0.6162	0.123416
rps7	0.189	-0.092	0.369	46.04	0.195	0.4	-0.58516	0.051613
ndhB	0.162	-0.094	0.349	46.43	0.279	0.375	0.644706	0.129412
ycf2	0.159	-0.136	0.338	52.8	0.345	0.377	-0.46346	0.123843
rpl23	0.115	-0.241	0.276	57.09	0.253	0.38	-0.42473	0.086022
rpl2	0.135	-0.117	0.346	50.84	0.293	0.435	-0.56716	0.04797
rpl2	0.148	-0.094	0.358	48.76	0.284	0.447	-0.54145	0.032895
rps19	0.187	-0.084	0.375	44.47	0.227	0.366	-0.5587	0.054348
