# Supplementary material for: The Complete Chloroplast Genome of Curcuma bakerii, an Endemic Medicinal Plant of Bangladesh: Insights into Genome Structure, Comparative Genomics, and Phylogenetic Relationships
Source: Genes (Basel). 2025 Dec 7;16(12):1460. doi: 10.3390/genes16121460 (PMC12732962; doi:10.3390/genes16121460)
Supplement: Supplementary file 1 [file genes-16-01460-s001.zip › Table S4.pdf]

Table S4: ΔRSCU and RFSC values of the codons in chloroplast genomes of Curcuma bakerii

Amino Acid	Codon	Count	RSCU high	Averge	RSCU low	ΔRSCU	RSFC	Amino Acid	Codon	Count	RSCU high	Averge	RSCU low	ΔRSCU	RSFC
Phe	UUU(F)	2414	1.24	1942.5	1.24273	-0.0027	0.62	Tyr	UAU(Y)	1720	1.37	1259	1.36616	0.0038364	0.68
	UUC(F)	1471	0.76	1942.5	0.75727	0.00273	0.38		UAC(Y)	798	0.63	1259	0.63384	-0.003836	0.32
Leu	UUA(L)	1177	1.34	876	1.34361	-0.0036	0.22	Stop	UAA(*)	1257	1.26	1013	1.24087	0.0191313	0.62
	UUG(L)	1087	1.24	876	1.24087	-0.0009	0.21		UAG(*)	769	0.77	1013	0.75913	0.0108687	0.38
	CUU(L)	1068	1.22	876	1.21918	0.00082	0.20	His	CAU(H)	958	1.4	686.5	1.39548	0.0045157	0.70
	CUC(L)	644	0.74	876	0.73516	0.00484	0.12		CAC(H)	415	0.6	686.5	0.60452	-0.004516	0.30
	CUA(L)	815	0.93	876	0.93037	-0.0004	0.16	Glu	CAA(Q)	1072	1.41	758	1.41425	-0.004248	0.71
	CUG(L)	465	0.53	876	0.53082	-0.0008	0.09		CAG(Q)	444	0.59	758	0.58575	0.004248	0.29
Iso	AUU(I)	1960	1.23	1594.6667	1.2291	0.0009	0.41	Asp	AAU(N)	1955	1.42	1379	1.41769	0.002306	0.71
	AUC(I)	1067	0.67	1594.6667	0.66911	0.00089	0.22		AAC(N)	803	0.58	1379	0.58231	-0.002306	0.29
	AUA(I)	1757	1.1	1594.6667	1.1018	-0.0018	0.37	Lys	AAA(K)	2304	1.37	1676.5	1.37429	-0.004292	0.69
Met	AUG(M)	944	1	944	1	0	1.00		AAG(K)	1049	0.63	1676.5	0.62571	0.0042917	0.31
Val	GUU(V)	788	1.29	609	1.29392	-0.0039	0.32	Aspartic	GAU(D)	1127	1.45	779	1.44673	0.0032734	0.72
	GUC(V)	469	0.77	609	0.77011	-0.0001	0.19253		GAC(D)	431	0.55	779	0.55327	-0.003273	0.28
	GUA(V)	776	1.27	609	1.27422	-0.0042	0.31856	Glutamic	GAA(E)	1411	1.41	1002.5	1.40748	0.0025187	0.70
	GUG(V)	403	0.66	609	0.66174	-0.0017	0.16544		GAG(E)	594	0.59	1002.5	0.59252	-0.002519	0.30
Ser	UCU(S)	1214	1.47	934.75	1.29874	0.17126	0.32469	Cys	UGU(C)	751	1.25	602	1.24751	0.0024917	0.62
	UCC(S)	936	1.13	934.75	1.00134	0.12866	0.25033		UGC(C)	453	0.75	602	0.75249	-0.002492	0.38
	UCA(S)	1001	1.21	934.75	1.07087	0.13913	0.26772	Stop	UGA(*)	965	0.97	965	1	-0.03	1.00
	UCG(S)	588	0.71	934.75	0.62905	0.08095	0.15726	Try	UGG(W)	690	1	690	1	0	1.00
Pro	CCU(P)	684	1.16	589.5	1.16031	-0.0003	0.29008	Arg	CGU(R)	399	0.72	399	1	-0.28	0.25
	CCC(P)	561	0.95	589.5	0.95165	-0.0017	0.23791		CGC(R)	227	0.41	399	0.56892	-0.158922	0.14

	CCA(P)	735	1.25	589.5	1.24682	0.00318	0.3117		CGA(R)	597	1.07	399	1.49624	-0.426241	0.37
	CCG(P)	378	0.64	589.5	0.64122	-0.0012	0.16031		CGG(R)	373	0.67	399	0.93484	-0.264837	0.23
Thr	ACU(T)	721	1.19	603.5	1.1947	-0.0047	0.29867	Ser	AGU(S)	747	0.9	609	1.2266	-0.326601	0.61
	ACC(T)	601	1	603.5	0.99586	0.00414	0.24896		AGC(S)	471	0.57	609	0.7734	-0.203399	0.39
	ACA(T)	728	1.21	603.5	1.2063	0.0037	0.30157	Arg	AGA(R)	1111	2	869.5	1.27775	0.7222542	0.64
	ACG(T)	364	0.6	603.5	0.60315	-0.0031	0.15079		AGG(R)	628	1.13	869.5	0.72225	0.4077458	0.36
Ala	GCU(A)	505	1.32	382.5	1.32026	-0.0003	0.33007	Gly	GGU(G)	591	1.08	549.5	1.07552	0.0044768	0.27
	GCC(A)	325	0.85	382.5	0.84967	0.00033	0.21242		GGC(G)	316	0.58	549.5	0.57507	0.0049318	0.14
	GCA(A)	477	1.25	382.5	1.24706	0.00294	0.31176		GGA(G)	805	1.46	549.5	1.46497	-0.004968	0.37
	GCG(A)	223	0.58	382.5	0.58301	-0.003	0.14575		GGG(G)	486	0.88	549.5	0.88444	-0.00444	0.22
