# Supplementary material for: The Complete Chloroplast Genome of Curcuma bakerii, an Endemic Medicinal Plant of Bangladesh: Insights into Genome Structure, Comparative Genomics, and Phylogenetic Relationships
Source: Genes (Basel). 2025 Dec 7;16(12):1460. doi: 10.3390/genes16121460 (PMC12732962; doi:10.3390/genes16121460)
Supplement: Supplementary file 1 [file genes-16-01460-s001.zip › Table S5.pdf]

Table S5: Long repeats position and location of Ten *Curcuma* Species

SN	Repeat Size	Repeat Position 1	Repeat Type	Repeat Location 1	Repeat Position 2	Repeat Location 2	E-Value
1	29750	87024	P	IGS	132439	ycf1	0.00E+00
2	54	30293	P	IGS	30293	IGS	2.28E-23
3	52	22	P	IGS	22	IGS	3.65E-22
4	44	125840	P	IGS	125840	IGS	2.39E-17
5	38	48618	P	IGS	48618	IGS	9.79E-14
6	37	48618	R	IGS	48618	IGS	3.92E-13
7	37	48618	C	IGS	48619	IGS	3.92E-13
8	37	48619	R	IGS	48619	IGS	3.92E-13
9	36	48618	P	IGS	48618	IGS	1.57E-12
10	36	48618	F	IGS	48620	IGS	1.57E-12
11	36	48620	P	IGS	48620	IGS	1.57E-12
12	35	48618	R	IGS	48618	IGS	6.27E-12
13	35	48618	C	IGS	48621	IGS	6.27E-12
14	35	48621	R	IGS	48621	IGS	6.27E-12
15	34	48618	P	IGS	48618	IGS	2.51E-11
16	34	48618	F	IGS	48622	IGS	2.51E-11
17	34	48622	P	IGS	48622	IGS	2.51E-11
18	34	112917	P	ycf1	112917	ycf1	2.51E-11
19	34	112917	F	ycf1	136262	ycf1	2.51E-11
20	34	136262	P	ycf1	136262	ycf1	2.51E-11
21	33	48618	R	IGS	48618	IGS	1.00E-10
22	33	48618	C	IGS	48623	IGS	1.00E-10
23	33	48623	R	IGS	48623	IGS	1.00E-10
24	32	38437	P	IGS	38479	IGS	4.01E-10
25	32	48618	P	IGS	48618	IGS	4.01E-10
26	32	48618	F	IGS	48624	IGS	4.01E-10
27	32	48624	P	IGS	48624	IGS	4.01E-10
28	31	29675	P	IGS	29727	IGS	1.60E-09
29	31	48589	R	IGS	48589	IGS	1.60E-09
30	31	48618	R	IGS	48618	IGS	1.60E-09
31	31	48618	C	IGS	48625	IGS	1.60E-09
32	31	48625	R	IGS	48625	IGS	1.60E-09
33	30	48618	P	IGS	48618	IGS	6.42E-09
34	30	48618	F	IGS	48626	IGS	6.42E-09
35	30	48626	P	IGS	48626	IGS	6.42E-09
36	29	13975	R	IGS	13975	IGS	2.57E-08
37	29	48618	R	IGS	48618	IGS	2.57E-08
38	29	48618	C	IGS	48627	IGS	2.57E-08
39	29	48627	R	IGS	48627	IGS	2.57E-08
40	28	48618	P	IGS	48618	IGS	1.03E-07
41	28	48618	F	IGS	48628	IGS	1.03E-07
42	28	48628	P	IGS	48628	IGS	1.03E-07

43	28	66383	P	IGS	66435	IGS	1.03E-07
44	27	30360	F	IGS	30376	IGS	4.11E-07
45	27	47939	P	IGS	53081	IGS	4.11E-07
46	27	48618	R	IGS	48618	IGS	4.11E-07
47	27	48618	C	IGS	48629	IGS	4.11E-07
48	27	48629	R	IGS	48629	IGS	4.11E-07
49	27	89719	F	trnI-CAU	89769	IGS	4.11E-07
50	27	89719	P	trnI-CAU	159417	IGS	4.11E-07
