# Supplementary material for: The Complete Chloroplast Genome of Curcuma bakerii, an Endemic Medicinal Plant of Bangladesh: Insights into Genome Structure, Comparative Genomics, and Phylogenetic Relationships
Source: Genes (Basel). 2025 Dec 7;16(12):1460. doi: 10.3390/genes16121460 (PMC12732962; doi:10.3390/genes16121460)
Supplement: Supplementary file 1 [file genes-16-01460-s001.zip › Table S6.pdf]

Table S6: The nucleotide diversity (Pi) value of ten Curcuma species

Start	End	Center	pi	Feature_names
1	600	300	0.001938	psbA|source
201	800	500	0.001259	psbA|source
401	1000	700	0.002963	psbA|source
601	1200	900	0.002963	psbA|source
801	1400	1100	0.003704	psbA|source
1001	1600	1300	0.004047	psbA|source|trnK-UUU
1201	1800	1500	0.004859	matK|source|trnK-UUU
1401	2000	1700	0.006047	matK|source|trnK-UUU
1601	2200	1900	0.004893	matK|source|trnK-UUU
1801	2400	2100	0.004868	matK|source|trnK-UUU
2001	2600	2300	0.002704	matK|source|trnK-UUU
2201	2800	2500	0.003111	matK|source|trnK-UUU
2401	3000	2700	0.002333	matK|source|trnK-UUU
2601	3200	2900	0.003222	matK|source|trnK-UUU
2801	3400	3100	0.001889	matK|source|trnK-UUU
3001	3600	3300	0.005025	matK|source|trnK-UUU
3201	3800	3500	0.004074	matK|source|trnK-UUU
3401	4000	3700	0.006267	source|trnK-UUU
3601	4200	3900	0.004272	source|trnK-UUU
3801	4400	4100	0.004294	source|trnK-UUU
4001	4600	4300	0.005575	source|trnK-UUU
4201	4800	4500	0.004606	source
4401	5000	4700	0.005468	rps16|source
4601	5200	4900	0.002503	rps16|source
4801	5400	5100	0.002165	rps16|source
5001	5600	5300	0.001449	rps16|source
5201	5800	5500	0.00413	rps16|source
5401	6000	5700	0.003762	rps16|source
5601	6200	5900	0.004854	rps16|source
5801	6400	6100	0.003055	rps16|source|trnQ-UUG
6001	6600	6300	0.004901	source|trnQ-UUG
6201	6800	6500	0.004167	psbK|source|trnQ-UUG
6401	7000	6700		psbK|source
6601	7200	6900		psbK|source
6801	7400	7100		psbK|source
7001	7600	7300	0.041975	psbI|source
7201	7800	7500	0.02423	psbI|source|trnS-GCU
7401	8000	7700	0.018114	psbI|source|trnS-GCU
7601	8200	7900	0.005525	source|trnS-GCU
7801	8400	8100	0.003845	source
8001	8600	8300	0.004618	source|trnG-UCC
8201	8800	8500	0.005536	source|trnG-UCC
8401	9000	8700	0.007317	source|trnG-UCC
8601	9200	8900	0.004986	source|trnG-UCC

8801	9400	9100	0.004454	source|trnG-UCC|trnR-UCU
9001	9600	9300	0.002302	atpA|source|trnG-UCC|trnR-UCU
9201	9800	9500	0.002633	atpA|source|trnG-UCC|trnR-UCU
9401	10000	9700	0.002245	atpA|source|trnR-UCU
9601	10200	9900	0.004308	atpA|source
9801	10400	10100	0.00636	atpA|source
10001	10600	10300	0.006697	atpA|source
10201	10800	10500	0.004852	atpA|source
10401	11000	10700	0.00381	atpA|source
10601	11200	10900	0.003361	atpA|atpF|source
10801	11400	11100	0.002689	atpA|atpF|source
11001	11600	11300	0.001778	atpA|atpF|source
11201	11800	11500	0.001889	atpF|source
11401	12000	11700	0.001556	atpF|source
11601	12200	11900	0.000778	atpF|source
11801	12400	12100	0.000333	atpF|source
12001	12600	12300	0.000333	atpF|source
12201	12800	12500	0.000667	atpF|source
12401	13000	12700	0.000667	atpF|atpH|source
12601	13200	12900	0.001347	atpH|source
12801	13400	13100	0.002894	atpH|source
13001	13600	13300	0.009043	atpH|source
13201	13800	13500	0.008606	atpH|source
13401	14000	13700	0.008718	source
13601	14200	13900	0.004199	source
13801	14400	14100	0.004781	source
14001	14600	14300	0.002912	source
14201	14800	14500	0.001489	atpI|source
14401	15000	14700	0.000668	atpI|source
14601	15200	14900	0.004368	atpI|source
14801	15400	15100	0.005848	atpI|source
15001	15600	15300	0.008238	atpI|source
15201	15800	15500	0.004762	atpI|rps2|source
15401	16000	15700	0.006782	atpI|rps2|source
15601	16200	15900	0.008436	rps2|source
15801	16400	16100	0.006739	rps2|source
16001	16600	16300	0.004138	rps2|source
16201	16800	16500	0.001375	rpoC2|rps2|source
16401	17000	16700	0.00099	rpoC2|rps2|source
16601	17200	16900	0.000667	rpoC2|source
16801	17400	17100	0.003691	rpoC2|source
17001	17600	17300	0.008352	rpoC2|source
17201	17800	17500	0.008008	rpoC2|source
17401	18000	17700	0.004847	rpoC2|source
17601	18200	17900	0.000333	rpoC2|source

17801	18400	18100	0.00179	rpoC2|source
18001	18600	18300	0.001852	rpoC2|source
18201	18800	18500	0.002546	rpoC2|source
18401	19000	18700	0.001724	rpoC2|source
18601	19200	18900	0.001667	rpoC2|source
18801	19400	19100	0.002111	rpoC2|source
19001	19600	19300	0.002594	rpoC2|source
19201	19800	19500	0.004174	rpoC2|source
19401	20000	19700	0.003647	rpoC2|source
19601	20200	19900	0.002815	rpoC2|source
19801	20400	20100	0.001259	rpoC2|source
20001	20600	20300	0.001778	rpoC2|source
20201	20800	20500	0.001778	rpoC2|source
20401	21000	20700	0.003481	rpoC1|rpoC2|source
20601	21200	20900	0.003037	rpoC1|rpoC2|source
20801	21400	21100	0.004593	rpoC1|rpoC2|source
21001	21600	21300	0.002556	rpoC1|source
21201	21800	21500	0.001889	rpoC1|source
21401	22000	21700	0.000333	rpoC1|source
21601	22200	21900	0.000333	rpoC1|source
21801	22400	22100	0.001519	rpoC1|source
22001	22600	22300	0.002556	rpoC1|source
22201	22800	22500	0.002889	rpoC1|source
22401	23000	22700	0.00263	rpoC1|source
22601	23200	22900	0.001259	rpoC1|source
22801	23400	23100	0.000926	rpoC1|source
23001	23600	23300	0.000333	rpoC1|source
23201	23800	23500	0.000333	rpoC1|source
23401	24000	23700	0.000333	rpoB|rpoC1|source
23601	24200	23900	0.000593	rpoB|rpoC1|source
23801	24400	24100	0.000593	rpoB|source
24001	24600	24300	0.00137	rpoB|source
24201	24800	24500	0.000778	rpoB|source
24401	25000	24700	0.001113	rpoB|source
24601	25200	24900	0.000668	rpoB|source
24801	25400	25100	0.000668	rpoB|source
25001	25600	25300	0.000926	rpoB|source
25201	25800	25500	0.000593	rpoB|source
25401	26000	25700	0.00137	rpoB|source
25601	26200	25900	0.001556	rpoB|source
25801	26400	26100	0.001889	rpoB|source
26001	26600	26300	0.002037	rpoB|source
26201	26800	26500	0.002037	rpoB|source
26401	27000	26700	0.00263	rpoB|source
26601	27200	26900	0.003074	rpoB|source
26801	27400	27100	0.002296	rpoB|source

27001	27600	27300	0.00137	rpoB|source
27201	27800	27500	0.000333	source
27401	28000	27700	0.001778	source
27601	28200	27900	0.003111	source|trnC-GCA
27801	28400	28100	0.003444	source|trnC-GCA
28001	28600	28300	0.003111	source|trnC-GCA
28201	28800	28500	0.001778	source|trnC-GCA
28401	29000	28700	0.00237	source
28601	29200	28900	0.002037	petN|source
28801	29400	29100	0.005746	petN|source
29001	29600	29300	0.005914	petN|source
29201	29800	29500	0.006275	source
29401	30000	29700	0.005013	source
29601	30200	29900	0.006071	source
29801	30400	30100	0.008751	source
30001	30600	30300	0.006453	psbM|source
30201	30800	30500	0.005469	psbM|source
30401	31000	30700	0.003126	psbM|source
30601	31200	30900	0.003596	psbM|source
30801	31400	31100	0.003629	source
31001	31600	31300	0.008588	source
31201	31800	31500	0.010923	source|trnD-GUC
31401	32000	31700	0.012018	source|trnD-GUC
31601	32200	31900	0.006876	source|trnD-GUC|trnY-GUA
31801	32400	32100	0.005786	source|trnE-UUC|trnY-GUA
32001	32600	32300	0.003798	source|trnE-UUC|trnY-GUA
32201	32800	32500	0.004122	source|trnE-UUC|trnY-GUA
32401	33000	32700	0.002515	source
32601	33200	32900	0.002363	source
32801	33400	33100	0.002301	source|trnT-GGU
33001	33600	33300	0.001962	source|trnT-GGU
33201	33800	33500	0.001669	source|trnT-GGU
33401	34000	33700	0.000705	source
33601	34200	33900	0.001373	source
33801	34400	34100	0.001815	source
34001	34600	34300	0.004493	source
34201	34800	34500	0.004205	psbD|source
34401	35000	34700	0.003364	psbD|source
34601	35200	34900	0.003223	psbD|source
34801	35400	35100	0.002867	psbD|source
35001	35600	35300	0.004037	psbD|source
35201	35800	35500	0.00101	psbC|psbD|source
35401	36000	35700	0.002923	psbC|psbD|source
35601	36200	35900	0.003131	psbC|psbD|source
35801	36400	36100	0.003529	psbC|source
36001	36600	36300	0.002049	psbC|source

36201	36800	36500	0.002323	psbC|source
36401	37000	36700	0.002264	psbC|source
36601	37200	36900	0.002222	psbC|source
36801	37400	37100	0.000667	psbC|source|trnS-UGA
37001	37600	37300	0.000333	psbC|source|trnS-UGA
37201	37800	37500	0.000333	psbZ|source|trnS-UGA
37401	38000	37700	0.000333	psbZ|source
37601	38200	37900	0.000667	psbZ|source|trnG-GCC
37801	38400	38100	0.001444	psbZ|source|trnG-GCC|trnfM-CAU
38001	38600	38300	0.002111	rps14|source|trnG-GCC|trnfM-CAU
38201	38800	38500	0.002111	rps14|source|trnfM-CAU
38401	39000	38700	0.001	rps14|source|trnfM-CAU
38601	39200	38900	0.005807	psaB|rps14|source
38801	39400	39100	0.006467	psaB|rps14|source
39001	39600	39300	0.007357	psaB|source
39201	39800	39500	0.002129	psaB|source
39401	40000	39700	0.001494	psaB|source
39601	40200	39900	0.000684	psaB|source
39801	40400	40100	0.000343	psaB|source
40001	40600	40300	0	psaB|source
40201	40800	40500	0.000948	psaB|source
40401	41000	40700	0.002519	psaB|source
40601	41200	40900	0.002519	psaB|source
40801	41400	41100	0.002185	psaA|psaB|source
41001	41600	41300	0.000593	psaA|psaB|source
41201	41800	41500	0.001185	psaA|psaB|source
41401	42000	41700	0.000593	psaA|source
41601	42200	41900	0.000593	psaA|source
41801	42400	42100	0	psaA|source
42001	42600	42300	0.000778	psaA|source
42201	42800	42500	0.000778	psaA|source
42401	43000	42700	0.001111	psaA|source
42601	43200	42900	0.000333	psaA|source
42801	43400	43100	0.000333	psaA|source
43001	43600	43300	0.000778	psaA|source
43201	43800	43500	0.001111	psaA|source
43401	44000	43700	0.001444	psaA|source
43601	44200	43900	0.001333	source|ycf3
43801	44400	44100	0.001333	source|ycf3
44001	44600	44300	0.001	source|ycf3
44201	44800	44500	0.001556	source|ycf3
44401	45000	44700	0.001222	source|ycf3
44601	45200	44900	0.001222	source|ycf3
44801	45400	45100	0.001259	source|ycf3
45001	45600	45300	0.001593	source|ycf3

45201	45800	45500	0.001601	source|ycf3
45401	46000	45700	0.00159	source|ycf3
45601	46200	45900	0.002637	source|ycf3
45801	46400	46100	0.002623	source|ycf3
46001	46600	46300	0.002263	source|ycf3
46201	46800	46500	0.001855	source|trnS-GGA
46401	47000	46700	0.004081	source|trnS-GGA
46601	47200	46900	0.003741	rps4|source|trnS-GGA
46801	47400	47100	0.003407	rps4|source|trnS-GGA
47001	47600	47300	0.002296	rps4|source
47201	47800	47500	0.001704	rps4|source
47401	48000	47700	0.001111	rps4|source
47601	48200	47900	0	rps4|source
47801	48400	48100	0.000342	source|trnT-UGU
48001	48600	48300	0.001497	source|trnT-UGU
48201	48800	48500	0.002397	source|trnT-UGU
48401	49000	48700	0.002041	source
48601	49200	48900	0.001496	source
48801	49400	49100	0.000599	source
49001	49600	49300	0.000593	source|trnL-UAA
49201	49800	49500	0.000778	source|trnL-UAA
49401	50000	49700	0.000778	source|trnL-UAA
49601	50200	49900	0.00289	source|trnL-UAA
49801	50400	50100	0.009947	source|trnF-GAA|trnL-UAA
50001	50600	50300	0.009947	source|trnF-GAA|trnL-UAA
50201	50800	50500	0.008253	source|trnF-GAA
50401	51000	50700	0.00737	source|trnF-GAA
50601	51200	50900	0.01327	ndhJ|source
50801	51400	51100	0.013138	ndhJ|source
51001	51600	51300	0.006725	ndhJ|ndhK|source
51201	51800	51500	0.003471	ndhJ|ndhK|source
51401	52000	51700	0.003153	ndhJ|ndhK|source
51601	52200	51900	0.002226	ndhK|source
51801	52400	52100	0.00137	ndhC|ndhK|source
52001	52600	52300	0.001365	ndhC|ndhK|source
52201	52800	52500	0.001002	ndhC|ndhK|source
52401	53000	52700	0.001026	ndhC|source
52601	53200	52900	0.002003	ndhC|source
52801	53400	53100	0.002841	source
53001	53600	53300	0.003039	source
53201	53800	53500	0.001721	source
53401	54000	53700	0.000929	source
53601	54200	53900	0.000334	source|trnV-UAC
53801	54400	54100	0.000334	source|trnV-UAC
54001	54600	54300	0.000926	source|trnV-UAC
54201	54800	54500	0.000593	source|trnV-UAC

54401	55000	54700	0.001259	source|trnV-UAC
54601	55200	54900	0.000667	source|trnM-CAU|trnV-UAC
54801	55400	55100	0.001351	atpE|source|trnM-CAU|trnV-UAC
55001	55600	55300	0.002696	atpE|source|trnM-CAU
55201	55800	55500	0.003494	atpB|atpE|source
55401	56000	55700	0.004053	atpB|atpE|source
55601	56200	55900	0.002652	atpB|atpE|source
55801	56400	56100	0.00198	atpB|source
56001	56600	56300	0.001539	atpB|source
56201	56800	56500	0.001023	atpB|source
56401	57000	56700	0.001102	atpB|source
56601	57200	56900	0	atpB|source
56801	57400	57100	0.00085	atpB|source
57001	57600	57300	0.000805	atpB|source
57201	57800	57500	0.001494	source
57401	58000	57700	0.001	rbcL|source
57601	58200	57900	0.002111	rbcL|source
57801	58400	58100	0.001778	rbcL|source
58001	58600	58300	0.002222	rbcL|source
58201	58800	58500	0.001444	rbcL|source
58401	59000	58700	0.002222	rbcL|source
58601	59200	58900	0.001778	rbcL|source
58801	59400	59100	0.001778	rbcL|source
59001	59600	59300	0.000667	rbcL|source
59201	59800	59500	0.000667	rbcL|source
59401	60000	59700	0.001008	rbcL|source
59601	60200	59900	0.001614	accD|source
59801	60400	60100	0.001979	accD|source
60001	60600	60300	0.002754	accD|source
60201	60800	60500	0.002486	accD|source
60401	61000	60700	0.002564	accD|source
60601	61200	60900	0.001115	accD|source
60801	61400	61100	0.000778	accD|source
61001	61600	61300	0.001815	accD|source
61201	61800	61500	0.002593	accD|source
61401	62000	61700	0.002593	accD|source
61601	62200	61900	0.001556	accD|source
61801	62400	62100	0.001452	psaI|source
62001	62600	62300	0.003438	psaI|source
62201	62800	62500	0.004628	psaI|source|ycf4
62401	63000	62700	0.004851	source|ycf4
62601	63200	62900	0.00339	source|ycf4
62801	63400	63100	0.001571	source|ycf4
63001	63600	63300	0.000673	source|ycf4
63201	63800	63500	0.000337	cemA|source|ycf4

63401	64000	63700	0.000333	cemA|source
63601	64200	63900	0.001444	cemA|source
63801	64400	64100	0.001111	cemA|source
64001	64600	64300	0.001111	cemA|source
64201	64800	64500	0	cemA|petA|source
64401	65000	64700	0.00195	cemA|petA|source
64601	65200	64900	0.007872	petA|source
64801	65400	65100	0.008164	petA|source
65001	65600	65300	0.007686	petA|source
65201	65800	65500	0.00263	petA|source
65401	66000	65700	0.002963	petA|source
65601	66200	65900	0.002444	petA|source
65801	66400	66100	0.005457	source
66001	66600	66300	0.006089	psbJ|source
66201	66800	66500	0.008106	psbJ|psbL|source
66401	67000	66700	0.004593	psbF|psbJ|psbL|source
66601	67200	66900	0.004111	psbE|psbF|psbJ|psbL|source
66801	67400	67100	0.000778	psbE|psbF|psbL|source
67001	67600	67300	0.002111	psbE|source
67201	67800	67500	0.004926	psbE|source
67401	68000	67700	0.005259	source
67601	68200	67900	0.004593	source
67801	68400	68100	0.002	source
68001	68600	68300	0.002333	petL|source
68201	68800	68500	0.004047	petG|petL|source
68401	69000	68700	0.003814	petG|petL|source
68601	69200	68900	0.004009	petG|source|trnW-CCA
68801	69400	69100	0.003191	petG|source|trnP-UGG|trnW-CCA
69001	69600	69300	0.007714	source|trnP-UGG|trnW-CCA
69201	69800	69500	0.006844	psaJ|source|trnP-UGG
69401	70000	69700	0.00522	psaJ|source
69601	70200	69900	0	psaJ|source
69801	70400	70100	0.001128	psaJ|source
70001	70600	70300	0.002256	rpl33|source
70201	70800	70500	0.002303	rpl33|source
70401	71000	70700	0.002582	rpl33|rps18|source
70601	71200	70900	0.001761	rpl33|rps18|source
70801	71400	71100	0.003333	rpl20|rps18|source
71001	71600	71300	0.003926	rpl20|rps18|source
71201	71800	71500	0.003926	rpl20|source
71401	72000	71700	0.002754	rpl20|source
71601	72200	71900	0.001377	rpl20|source
71801	72400	72100	0.00218	source
72001	72600	72300	0.003275	rps12|source
72201	72800	72500	0.004498	clpP1|rps12|source

72401	73000	72700	0.003345	clpP1|rps12|source
72601	73200	72900	0.001895	clpP1|rps12|source
72801	73400	73100	0.002443	clpP1|rps12|source
73001	73600	73300	0.006243	clpP1|rps12|source
73201	73800	73500	0.006221	clpP1|rps12|source
73401	74000	73700	0.004676	clpP1|rps12|source
73601	74200	73900	0.002367	clpP1|rps12|source
73801	74400	74100	0.002711	clpP1|rps12|source
74001	74600	74300	0.005685	clpP1|rps12|source
74201	74800	74500	0.006003	clpP1|rps12|source
74401	75000	74700	0.005645	clpP1|rps12|source
74601	75200	74900	0.002263	clpP1|rps12|source
74801	75400	75100	0.000594	clpP1|psbB|rps12|source
75001	75600	75300	0.001721	psbB|rps12|source
75201	75800	75500	0.002577	psbB|rps12|source
75401	76000	75700	0.00361	psbB|rps12|source
75601	76200	75900	0.002461	psbB|rps12|source
75801	76400	76100	0.001406	psbB|rps12|source
76001	76600	76300	0.000364	psbB|rps12|source
76201	76800	76500	0.00162	psbB|rps12|source
76401	77000	76700	0.001905	psbB|rps12|source
76601	77200	76900	0.002511	psbB|psbN|psbT|rps12|source
76801	77400	77100	0.002013	psbB|psbN|psbT|rps12|source
77001	77600	77300	0.001354	psbH|psbN|psbT|rps12|source
77201	77800	77500	0.001087	petB|psbH|psbN|rps12|source
77401	78000	77700	0.002046	petB|psbH|rps12|source
77601	78200	77900	0.00202	petB|psbH|rps12|source
77801	78400	78100	0.004323	petB|rps12|source
78001	78600	78300	0.003413	petB|rps12|source
78201	78800	78500	0.00378	petB|rps12|source
78401	79000	78700	0.001678	petB|rps12|source
78601	79200	78900	0.00179	petB|rps12|source
78801	79400	79100	0.002222	petB|rps12|source
79001	79600	79300	0.002333	petB|petD|rps12|source
79201	79800	79500	0.001889	petB|petD|rps12|source
79401	80000	79700	0.001778	petD|rps12|source
79601	80200	79900	0.002222	petD|rps12|source
79801	80400	80100	0.001889	petD|rps12|source
80001	80600	80300	0.001815	petD|rps12|source
80201	80800	80500	0.001185	petD|rps12|source
80401	81000	80700	0.001519	petD|rpoA|rps12|source
80601	81200	80900	0.001614	petD|rpoA|rps12|source
80801	81400	81100	0.002034	rpoA|rps12|source
81001	81600	81300	0.001695	rpoA|rps12|source
81201	81800	81500	0.001361	rpoA|rps12|source
81401	82000	81700	0.000943	rpoA|rps12|source

81601	82200	81900	0.000943	rpoA|rps11|rps12|source
81801	82400	82100	0.000927	rpoA|rps11|rps12|source
82001	82600	82300	0.001111	rpl36|rps11|rps12|source
82201	82800	82500	0.001444	rpl36|rps11|rps12|source
82401	83000	82700	0.001111	infA|rpl36|rps11|rps12|source
82601	83200	82900	0.000337	infA|rpl36|rps12|rps8|source
82801	83400	83100	0.001124	infA|rps12|rps8|source
83001	83600	83300	0.001124	infA|rps12|rps8|source
83201	83800	83500	0.001778	rpl14|rps12|rps8|source
83401	84000	83700	0.001	rpl14|rps12|rps8|source
83601	84200	83900	0.001333	rpl14|rps12|source
83801	84400	84100	0.004444	rpl14|rpl16|rps12|source
84001	84600	84300	0.004103	rpl14|rpl16|rps12|source
84201	84800	84500	0.003761	rpl16|rps12|source
84401	85000	84700	0	rpl16|rps12|source
84601	85200	84900	0.000333	rpl16|rps12|source
84801	85400	85100	0.001753	rpl16|rps12|source
85001	85600	85300	0.002897	rpl16|rps12|source
85201	85800	85500	0.002897	rpl16|rps12|source
85401	86000	85700	0.001781	rpl16|rps12|rps3|source
85601	86200	85900	0.001002	rpl16|rps12|rps3|source
85801	86400	86100	0.001595	rps12|rps3|source
86001	86600	86300	0.001266	rpl22|rps12|rps3|source
86201	86800	86500	0.000931	rpl22|rps12|rps3|source
86401	87000	86700	0.000672	rpl22|rps12|rps3|source
86601	87200	86900	0.000712	rpl22|rps12|rps19|source
86801	87400	87100	0.001898	rpl22|rps12|rps19|source
87001	87600	87300	0.001537	rps12|rps19|source|trnH-GUG
87201	87800	87500	0.003	rpl2|rps12|rps19|source|trnH-GUG
87401	88000	87700	0.002815	rpl2|rps12|rps19|source|trnH-GUG
87601	88200	87900	0.002481	rpl2|rps12|source|trnH-GUG
87801	88400	88100	0.002824	rpl2|rps12|source
88001	88600	88300	0.003419	rpl2|rps12|source
88201	88800	88500	0.004013	rpl2|rps12|source
88401	89000	88700	0.002889	rpl2|rps12|source
88601	89200	88900	0.001721	rpl2|rps12|source
88801	89400	89100	0.002507	rpl2|rpl23|rps12|source
89001	89600	89300	0.001721	rpl2|rpl23|rps12|source
89201	89800	89500	0.003074	rpl23|rps12|source|trnI-CAU
89401	90000	89700	0.001704	rpl23|rps12|source|trnI-CAU|ycf2
89601	90200	89900	0.002815	rps12|source|trnI-CAU|ycf2
89801	90400	90100	0.002889	rps12|source|ycf2
90001	90600	90300	0.017655	rps12|source|ycf2

90201	90800	90500	0.016432	rps12|source|ycf2
90401	91000	90700	0.014869	rps12|source|ycf2
90601	91200	90900	0.001526	rps12|source|ycf2
90801	91400	91100	0.001861	rps12|source|ycf2
91001	91600	91300	0.001524	rps12|source|ycf2
91201	91800	91500	0.000333	rps12|source|ycf2
91401	92000	91700	0	rps12|source|ycf2
91601	92200	91900	0.000333	rps12|source|ycf2
91801	92400	92100	0.000333	rps12|source|ycf2
92001	92600	92300	0.000333	rps12|source|ycf2
92201	92800	92500	0	rps12|source|ycf2
92401	93000	92700	0	rps12|source|ycf2
92601	93200	92900	0	rps12|source|ycf2
92801	93400	93100	0	rps12|source|ycf2
93001	93600	93300	0	rps12|source|ycf2
93201	93800	93500	0	rps12|source|ycf2
93401	94000	93700	0.000333	rps12|source|ycf2
93601	94200	93900	0.000333	rps12|source|ycf2
93801	94400	94100	0.000667	rps12|source|ycf2
94001	94600	94300	0.000333	rps12|source|ycf2
94201	94800	94500	0.000333	rps12|source|ycf2
94401	95000	94700	0	rps12|source|ycf2
94601	95200	94900	0.000667	rps12|source|ycf2
94801	95400	95100	0.001	rps12|source|ycf2
95001	95600	95300	0.001	rps12|source|ycf2
95201	95800	95500	0.000333	rps12|source|ycf2
95401	96000	95700	0	rps12|source|ycf2
95601	96200	95900	0.000333	rps12|source|ycf2
95801	96400	96100	0.000333	rps12|source|ycf2
96001	96600	96300	0.000333	rps12|source|ycf2
96201	96800	96500	0	rps12|source|ycf2
96401	97000	96700	0	rps12|source|ycf2
96601	97200	96900	0.000778	rps12|source|ycf2
96801	97400	97100	0.000778	rps12|source
97001	97600	97300	0.000778	rps12|source
97201	97800	97500	0	rps12|source|trnL-CAA
97401	98000	97700	0	rps12|source|trnL-CAA
97601	98200	97900	0	rps12|source|trnL-CAA
97801	98400	98100	0.000926	ndhB|rps12|source
98001	98600	98300	0.000926	ndhB|rps12|source
98201	98800	98500	0.000926	ndhB|rps12|source
98401	99000	98700	0	ndhB|rps12|source
98601	99200	98900	0	ndhB|rps12|source
98801	99400	99100	0.000333	ndhB|rps12|source
99001	99600	99300	0.000333	ndhB|rps12|source
99201	99800	99500	0.000333	ndhB|rps12|source

99401	100000	99700	0	ndhB|rps12|source
99601	100200	99900	0	ndhB|rps12|source
99801	100400	100100	0.001	ndhB|rps12|source
100001	100600	100300	0.001333	ndhB|rps12|source
100201	100800	100500	0.001333	ndhB|rps12|rps7|source
100401	101000	100700	0.000667	ndhB|rps12|rps7|source
100601	101200	100900	0.000667	rps12|rps7|source
100801	101400	101100	0.001008	rps12|rps7|source
101001	101600	101300	0.000672	rps12|rps7|source
101201	101800	101500	0.000336	rps12|rps7|source
101401	102000	101700	0	rps12|source
101601	102200	101900	0.000333	rps12|source
101801	102400	102100	0.001111	rps12|source
102001	102600	102300	0.001111	rps12|source
102201	102800	102500	0.000778	rps12|source
102401	103000	102700	0	rps12|source
102601	103200	102900	0.000926	rps12|source
102801	103400	103100	0.000926	rps12|source
103001	103600	103300	0.000926	rps12|source
103201	103800	103500	0	rps12|source
103401	104000	103700	0	rps12|source
103601	104200	103900	0	rps12|source|trnV-GAC
103801	104400	104100	0	rps12|rrn16|source|trnV-GAC
104001	104600	104300	0	rps12|rrn16|source|trnV-GAC
104201	104800	104500	0	rps12|rrn16|source
104401	105000	104700	0	rps12|rrn16|source
104601	105200	104900	0	rps12|rrn16|source
104801	105400	105100	0	rps12|rrn16|source
105001	105600	105300	0	rps12|rrn16|source
105201	105800	105500	0	rps12|rrn16|source
105401	106000	105700	0.000937	rps12|rrn16|source
105601	106200	105900	0.001724	rps12|rrn16|source|trnI-GAU
105801	106400	106100	0.001724	rps12|rrn16|source|trnI-GAU
106001	106600	106300	0.001136	rps12|source|trnI-GAU
106201	106800	106500	0.000341	rps12|source|trnI-GAU
106401	107000	106700	0.000355	rps12|source|trnI-GAU
106601	107200	106900	0.000347	rps12|source|trnA-UGC|trnI-GAU
106801	107400	107100	0.000693	rps12|source|trnA-UGC|trnI-GAU
107001	107600	107300	0.000667	rps12|source|trnA-UGC|trnI-GAU
107201	107800	107500	0.000667	rps12|source|trnA-UGC
107401	108000	107700	0.000333	rps12|source|trnA-UGC
107601	108200	107900	0.000333	rps12|source|trnA-UGC

107801	108400	108100	0	rps12|rrn23|source|trnA-UGC
108001	108600	108300	0	rps12|rrn23|source|trnA-UGC
108201	108800	108500	0	rps12|rrn23|source
108401	109000	108700	0.001111	rps12|rrn23|source
108601	109200	108900	0.001111	rps12|rrn23|source
108801	109400	109100	0.001111	rps12|rrn23|source
109001	109600	109300	0	rps12|rrn23|source
109201	109800	109500	0	rps12|rrn23|source
109401	110000	109700	0	rps12|rrn23|source
109601	110200	109900	0	rps12|rrn23|source
109801	110400	110100	0	rps12|rrn23|source
110001	110600	110300	0	rps12|rrn23|source
110201	110800	110500	0.000778	rps12|rrn23|source
110401	111000	110700	0.000778	rps12|rrn23|source
110601	111200	110900	0.001111	rps12|rrn23|rrn4.5|source
110801	111400	111100	0.000667	rps12|rrn23|rrn4.5|source
111001	111600	111300	0.000667	rps12|rrn4.5|rrn5|source
111201	111800	111500	0.000667	rps12|rrn4.5|rrn5|source
111401	112000	111700	0.001111	rps12|rrn5|source|trnR-ACG
111601	112200	111900	0.001111	rps12|source|trnR-ACG
111801	112400	112100	0.000778	rps12|source|trnR-ACG
112001	112600	112300	0	rps12|source|trnN-GUU
112201	112800	112500	0.000779	rps12|source|trnN-GUU
112401	113000	112700	0.000779	rps12|source|trnN-GUU|ycf1
112601	113200	112900	0.000779	rps12|source|ycf1
112801	113400	113100	0	rps12|source|ycf1
113001	113600	113300	0	rps12|source|ycf1
113201	113800	113500	0	rps12|source|ycf1
113401	114000	113700	0.000333	rps12|source|ycf1
113601	114200	113900	0.000667	rps12|source|ycf1
113801	114400	114100	0.000667	rps12|source|ycf1
114001	114600	114300	0.000667	rps12|source|ycf1
114201	114800	114500	0.000333	rps12|source|ycf1
114401	115000	114700	0.000333	rps12|source|ycf1
114601	115200	114900	0.000926	rps12|source|ycf1
114801	115400	115100	0.000926	rps12|source|ycf1
115001	115600	115300	0.001259	rps12|source|ycf1
115201	115800	115500	0.000667	rps12|source|ycf1
115401	116000	115700	0.000667	rps12|source|ycf1
115601	116200	115900	0.000333	rps12|source|ycf1
115801	116400	116100	0.000778	rps12|source|ycf1
116001	116600	116300	0.000778	rps12|source|ycf1
116201	116800	116500	0.000778	ndhF|rps12|source|ycf1
116401	117000	116700	0	ndhF|rps12|source|ycf1
116601	117200	116900	0	ndhF|rps12|source|ycf1

116801	117400	117100	0	ndhF|rps12|source|ycf1
117001	117600	117300	0.000333	ndhF|rps12|source
117201	117800	117500	0.001111	ndhF|rps12|source
117401	118000	117700	0.001444	ndhF|rps12|source
117601	118200	117900	0.002	ndhF|rps12|source
117801	118400	118100	0.001222	ndhF|rps12|source
118001	118600	118300	0.000889	ndhF|rps12|source
118201	118800	118500	0	ndhF|rps12|source
118401	119000	118700	0.000333	ndhF|rps12|source
118601	119200	118900	0.000333	ndhF|rps12|source
118801	119400	119100	0.001111	ndhF|rps12|source
119001	119600	119300	0.000778	ndhF|rps12|source
119201	119800	119500	0.000778	rpl32|rps12|source
119401	120000	119700	0	rpl32|rps12|source
119601	120200	119900	0	rpl32|rps12|source
119801	120400	120100	0	rpl32|rps12|source
120001	120600	120300	0.005556	rps12|source
120201	120800	120500	0.015716	rps12|source|trnL-UAG
120401	121000	120700	0.009748	ccsA|rps12|source|trnL-UAG
120601	121200	120900	0.006693	ccsA|rps12|source|trnL-UAG
120801	121400	121100	0.005778	ccsA|rps12|source
121001	121600	121300	0.006778	ccsA|rps12|source
121201	121800	121500	0.004778	ccsA|rps12|source
121401	122000	121700	0.002	ccsA|rps12|source
121601	122200	121900	0.002259	ccsA|ndhD|rps12|source
121801	122400	122100	0.002926	ccsA|ndhD|rps12|source
122001	122600	122300	0.001926	ndhD|rps12|source
122201	122800	122500	0.001593	ndhD|rps12|source
122401	123000	122700	0.003143	ndhD|rps12|source
122601	123200	122900	0.005641	ndhD|rps12|source
122801	123400	123100	0.011866	ndhD|rps12|source
123001	123600	123300	0.013114	ndhD|rps12|source
123201	123800	123500	0.011813	ndhD|psaC|rps12|source
123401	124000	123700	0.008414	ndhD|psaC|rps12|source
123601	124200	123900	0.006089	psaC|rps12|source
123801	124400	124100	0.007361	psaC|rps12|source
124001	124600	124300	0.004297	psaC|rps12|source
124201	124800	124500	0.004644	ndhE|rps12|source
124401	125000	124700	0.003561	ndhE|rps12|source
124601	125200	124900	0.004118	ndhE|ndhG|rps12|source
124801	125400	125100	0.005333	ndhE|ndhG|rps12|source
125001	125600	125300	0.004148	ndhG|rps12|source
125201	125800	125500	0.003407	ndhG|rps12|source
125401	126000	125700	0.005785	ndhG|rps12|source
125601	126200	125900	0.007659	ndhG|ndhI|rps12|source
125801	126400	126100	0.007738	ndhI|rps12|source

126001	126600	126300	0.004023	ndhI|rps12|source
126201	126800	126500	0.002778	ndhA|ndhI|rps12|source
126401	127000	126700	0.002704	ndhA|ndhI|rps12|source
126601	127200	126900	0.00137	ndhA|ndhI|rps12|source
126801	127400	127100	0.001593	ndhA|rps12|source
127001	127600	127300	0.001	ndhA|rps12|source
127201	127800	127500	0.001333	ndhA|rps12|source
127401	128000	127700	0.000333	ndhA|rps12|source
127601	128200	127900	0.002423	ndhA|rps12|source
127801	128400	128100	0.003416	ndhA|rps12|source
128001	128600	128300	0.010782	ndhA|rps12|source
128201	128800	128500	0.011998	ndhA|rps12|source
128401	129000	128700	0.011068	ndhA|ndhH|rps12|source
128601	129200	128900	0.004436	ndhA|ndhH|rps12|source
128801	129400	129100	0.002574	ndhA|ndhH|rps12|source
129001	129600	129300	0.002328	ndhH|rps12|source
129201	129800	129500	0.004044	ndhH|rps12|source
129401	130000	129700	0.004497	ndhH|rps12|source
129601	130200	129900	0.004719	ndhH|rps12|rps15|source
129801	130400	130100	0.003146	ndhH|rps12|rps15|source
130001	130600	130300	0.001912	ndhH|rps12|rps15|source
130201	130800	130500	0.001111	rps12|rps15|source
130401	131000	130700	0.001667	rps12|rps15|source|ycf1
130601	131200	130900	0.002	rps12|source|ycf1
130801	131400	131100	0.002	rps12|source|ycf1
131001	131600	131300	0.000671	rps12|source|ycf1
131201	131800	131500	0.001014	rps12|source|ycf1
131401	132000	131700	0.002431	rps12|source|ycf1
131601	132200	131900	0.007446	rps12|source|ycf1
131801	132400	132100	0.006712	rps12|source|ycf1
132001	132600	132300	0.005961	rps12|source|ycf1
132201	132800	132500	0.003691	rps12|source|ycf1
132401	133000	132700	0.004474	rps12|source|ycf1
132601	133200	132900	0.005	rps12|source|ycf1
132801	133400	133100	0.002333	rps12|source|ycf1
133001	133600	133300	0.002667	rps12|source|ycf1
133201	133800	133500	0.001444	rps12|source|ycf1
133401	134000	133700	0.002222	rps12|source|ycf1
133601	134200	133900	0.000778	rps12|source|ycf1
133801	134400	134100	0.001425	rps12|source|ycf1
134001	134600	134300	0.003428	rps12|source|ycf1
134201	134800	134500	0.005181	rps12|source|ycf1
134401	135000	134700	0.006506	rps12|source|ycf1
134601	135200	134900	0.003998	rps12|source|ycf1
134801	135400	135100	0.004482	rps12|source|ycf1
135001	135600	135300	0.004704	rps12|source|ycf1

135201	135800	135500	0.00563	rps12|source|ycf1
135401	136000	135700	0.005222	rps12|source|ycf1
135601	136200	135900	0.004519	rps12|source|ycf1
135801	136400	136100	0.007407	rps12|source|ycf1
136001	136600	136300	0.00873	rps12|source|ycf1
136201	136800	136500	0.009758	rps12|source|trnN-GUU|ycf1
136401	137000	136700	0.00571	rps12|source|trnN-GUU
136601	137200	136900	0.002585	rps12|source|trnN-GUU
136801	137400	137100	0	rps12|source|trnR-ACG
137001	137600	137300	0	rps12|source|trnR-ACG
137201	137800	137500	0.000778	rps12|rrn5|source|trnR-ACG
137401	138000	137700	0.000778	rps12|rrn4.5|rrn5|source
137601	138200	137900	0.001111	rps12|rrn4.5|rrn5|source
137801	138400	138100	0.000333	rps12|rrn23|rrn4.5|source
138001	138600	138300	0.000333	rps12|rrn23|rrn4.5|source
138201	138800	138500	0	rps12|rrn23|source
138401	139000	138700	0.000889	rps12|rrn23|source
138601	139200	138900	0.001222	rps12|rrn23|source
138801	139400	139100	0.002	rps12|rrn23|source
139001	139600	139300	0.001444	rps12|rrn23|source
139201	139800	139500	0.001111	rps12|rrn23|source
139401	140000	139700	0.000333	rps12|rrn23|source
139601	140200	139900	0	rps12|rrn23|source
139801	140400	140100	0	rps12|rrn23|source
140001	140600	140300	0.000778	rps12|rrn23|source
140201	140800	140500	0.000778	rps12|rrn23|source
140401	141000	140700	0.000778	rps12|rrn23|source
140601	141200	140900	0	rps12|rrn23|source|trnA-UGC
140801	141400	141100	0.000333	rps12|rrn23|source|trnA-UGC
141001	141600	141300	0.000667	rps12|rrn23|source|trnA-UGC
141201	141800	141500	0.000667	rps12|source|trnA-UGC
141401	142000	141700	0.001259	rps12|source|trnA-UGC
141601	142200	141900	0.000926	rps12|source|trnA-UGC|trnI-GAU
141801	142400	142100	0.000926	rps12|source|trnA-UGC|trnI-GAU
142001	142600	142300	0	rps12|source|trnA-UGC|trnI-GAU
142201	142800	142500	0	rps12|source|trnI-GAU
142401	143000	142700	0	rps12|source|trnI-GAU
142601	143200	142900	0	rps12|source|trnI-GAU
142801	143400	143100	0.000333	rps12|source|trnI-GAU
143001	143600	143300	0.000333	rps12|rrn16|source|trnI-GAU
143201	143800	143500	0.000333	rps12|rrn16|source

143401	144000	143700	0	rps12|rrn16|source
143601	144200	143900	0	rps12|rrn16|source
143801	144400	144100	0.000779	rps12|rrn16|source
144001	144600	144300	0.000779	rps12|rrn16|source
144201	144800	144500	0.000779	rps12|rrn16|source
144401	145000	144700	0	rps12|rrn16|source
144601	145200	144900	0.001111	rps12|rrn16|source|trnV-GAC
144801	145400	145100	0.001111	rps12|rrn16|source|trnV-GAC
145001	145600	145300	0.001111	rps12|source|trnV-GAC
145201	145800	145500	0.000333	rps12|source
145401	146000	145700	0.000667	rps12|source
145601	146200	145900	0.000667	rps12|source
145801	146400	146100	0.001111	rps12|source
146001	146600	146300	0.000778	rps12|source
146201	146800	146500	0.000778	rps12|source
146401	147000	146700	0	rps12|source
146601	147200	146900	0	rps12|source
146801	147400	147100	0	rps12|source
147001	147600	147300	0	rps12|source
147201	147800	147500	0	rps12|source
147401	148000	147700	0	rps12|rps7|source
147601	148200	147900	0.001111	rps12|rps7|source
147801	148400	148100	0.001111	rps12|rps7|source
148001	148600	148300	0.001111	rps7|source
148201	148800	148500	0	ndhB|rps7|source
148401	149000	148700	0	ndhB|rps7|source
148601	149200	148900	0.000333	ndhB|source
148801	149400	149100	0.000333	ndhB|source
149001	149600	149300	0.000333	ndhB|source
149201	149800	149500	0.000333	ndhB|source
149401	150000	149700	0.000667	ndhB|source
149601	150200	149900	0.000693	ndhB|source
149801	150400	150100	0.000355	ndhB|source
150001	150600	150300	0.000355	ndhB|source
150201	150800	150500	0.000341	ndhB|source
150401	151000	150700	0.001126	ndhB|source
150601	151200	150900	0.001727	ndhB|source
150801	151400	151100	0.001727	ndhB|source
151001	151600	151300	0.000926	source|trnL-CAA
151201	151800	151500	0	source|trnL-CAA
151401	152000	151700	0	source|trnL-CAA
151601	152200	151900	0	source
151801	152400	152100	0	source
152001	152600	152300	0	source|ycf2
152201	152800	152500	0	source|ycf2

152401	153000	152700	0	source|ycf2
152601	153200	152900	0	source|ycf2
152801	153400	153100	0	source|ycf2
153001	153600	153300	0	source|ycf2
153201	153800	153500	0.000333	source|ycf2
153401	154000	153700	0.000926	source|ycf2
153601	154200	153900	0.000926	source|ycf2
153801	154400	154100	0.000593	source|ycf2
154001	154600	154300	0.000778	source|ycf2
154201	154800	154500	0.000778	source|ycf2
154401	155000	154700	0.001111	source|ycf2
154601	155200	154900	0.000333	source|ycf2
154801	155400	155100	0.000333	source|ycf2
155001	155600	155300	0.000336	source|ycf2
155201	155800	155500	0.000336	source|ycf2
155401	156000	155700	0.000672	source|ycf2
155601	156200	155900	0.000667	source|ycf2
155801	156400	156100	0.000667	source|ycf2
156001	156600	156300	0.001	source|ycf2
156201	156800	156500	0.001333	source|ycf2
156401	157000	156700	0.001333	source|ycf2
156601	157200	156900	0.000667	source|ycf2
156801	157400	157100	0	source|ycf2
157001	157600	157300	0	source|ycf2
157201	157800	157500	0.000333	source|ycf2
157401	158000	157700	0.000333	source|ycf2
157601	158200	157900	0.000333	source|ycf2
157801	158400	158100	0	source|ycf2
158001	158600	158300	0	source|ycf2
158201	158800	158500	0.000926	source|ycf2
158401	159000	158700	0.000926	source|ycf2
158601	159200	158900	0.000926	source|ycf2
158801	159400	159100	0	source|ycf2
159001	159600	159300	0	source|trnI-CAU|ycf2
159201	159800	159500	0.000778	rpl23|source|trnI-CAU|ycf2
159401	160000	159700	0.000778	rpl23|source|trnI-CAU
159601	160200	159900	0.000778	rpl2|rpl23|source
159801	160400	160100	0	rpl2|rpl23|source
160001	160600	160300	0	rpl2|rpl23|source
160201	160800	160500	0	rpl2|source
160401	161000	160700	0.000333	rpl2|source
160601	161200	160900	0.000333	rpl2|source
160801	161400	161100	0.000333	rpl2|source
161001	161600	161300	0	rpl2|source|trnH-GUG
161201	161800	161500	0.000667	rpl2|rps19|source|trnH-GUG
161401	162000	161700	0.001	rpl2|rps19|source|trnH-GUG

161601	162200	161900	0.001	rps19|source|trnH-GUG
161801	162400	162100	0.000333	rps19|source
162001	162600	162300	0	rps19|source
162201	162800	162500	0.000333	intergenic
162401	163000	162700	0.000667	intergenic
162601	163200	162900	0.000667	intergenic
162801	163400	163100	0.000333	intergenic
163001	163600	163300	0	intergenic
163201	163800	163500	0	intergenic
163401	164000	163700	0	intergenic
163601	164200	163900	0	intergenic
163801	164400	164100	0	intergenic
164001	164600	164300	0	intergenic
164201	164800	164500	0.000333	intergenic
164401	165000	164700	0.000333	intergenic
164601	165200	164900	0.000333	intergenic
164801	165400	165100	0	intergenic
165001	165600	165300	0.000333	intergenic
165201	165800	165500	0.000333	intergenic
165401	166000	165700	0.001709	intergenic
165601	166200	165900	0.001712	intergenic
165801	166400	166100	0.001736	intergenic
166001	166570	166285	0.007633	intergenic
